# Dynamic microenvironment-regulated hydrogels releasing celastrol lead to urethral scarless repair

**DOI:** 10.1016/j.mtbio.2026.102917

**Published:** 2026-02-11

**Authors:** Yangwang Jin, Fei Qin, Ranxing Yang, Wenzhuo Fang, Kaile Zhang, Meng Liu, Ming Yang, Ying Wang, Qiang Fu

**Affiliations:** a Department of Urology, Shanghai Sixth People's Hospital Affiliated to Shanghai Jiao Tong University School of Medicine, Shanghai Eastern Institute of Urologic Reconstruction, Shanghai Jiao Tong University, Shanghai, 200233, China; b Department of Urology, Wuxi 9th People's Hospital, 999 Liangxi Road, Binhu District, Wuxi, Jiangsu Province, 214061, China

**Keywords:** Pathological microenvironment, Response hydrogel, Fibroblast activation, Celastrol delivery, Scarless urethral reconstruction

## Abstract

The dynamic pathological microenvironment formed post-urethral injury drives an inflammatory-fibrotic cascade, leading to urethral stricture. Herein, we designed a novel hydrogel dressing (CPT-Cel) to modulate this dynamic urethral microenvironment for scarless reconstruction. The hydrogel responsively degrades and releases its contents upon encountering acidic environments and ROS bursts, interrupting the severe oxidative stress-inflammation cycle and creating a favorable regenerative microenvironment in the early wound phase. The incorporated celastrol continuously releases into the late repair phase, inhibiting the hyperactivation of urethral fibroblasts via the TGF-β/NF-κB pathway in urethral microenviroment, thereby preventing excessive collagen secretion. Furthermore, the dressing, featuring an ECM-mimicking microstructure, effectively adheres to and integrates with the injury site, providing hemostatic and antibacterial functions. In animal models of urethral injury, CPT-Cel accelerated urethral repair within the inflammatory milieu, significantly reduced collagen deposition and ultimately improved stricture outcomes. This study not only validates the core mechanism by which an intelligent drug-loaded hydrogel modulates the dynamic urethral microenvironment but also proposes a new paradigm for urethral tissue engineering.

## Graphical abstract

**Figure:**
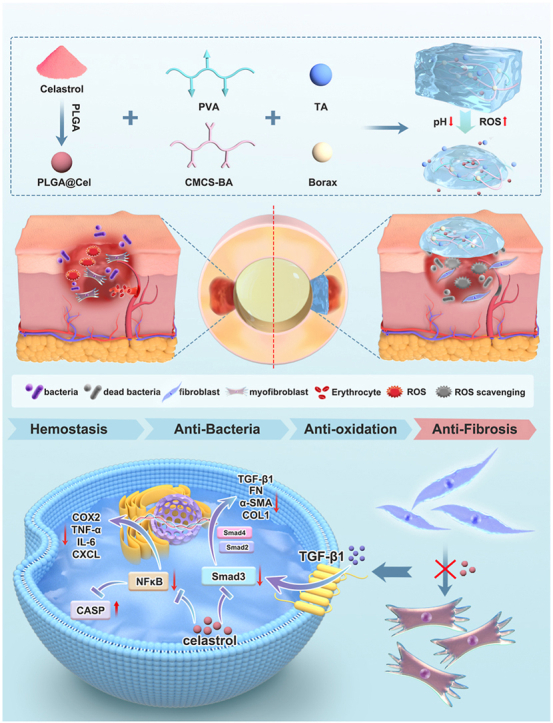

## 1 Introduction

Urethral injury is a common and complex clinical condition that often leads to excessive collagen deposition and tissue fibrosis, resulting in urethral scarring, which ultimately progresses to urethral stricture and even complete obstruction [[1], [2], [3], [4], [5]]. Tissue-engineered materials, particularly hydrogels, offer a promising alternative and have demonstrated significant potential in regenerative medicine [[6], [7], [8], [9]]. Nevertheless, research dedicated to urethral repair and reconstruction remains scarce, with a critical lack of effective strategies to promote scarless urethral healing for clinical translation [10].

The pathological basis of post-traumatic urethral scarring lies in an imbalanced inflammatory-fibrotic cascade driven by the uniquely hostile urethral microenvironment. The deterioration of the urethral microenvironment in the early stage of urethral injury or the passage of urine not only induces inflammatory cell aggregation and excessive production of reactive oxygen species (ROS), which leads to oxidative stress and even cell death, but also aggravates the acidic environment of the wound, which affects cellular activity, proliferation, and migration [[11], [12], [13]]. In addition, bacteria in the urine, such as *Escherichia coli*, often cause infections that can lead to tissue necrosis and severe complications [14]. The combination of these factors diminishes cell proliferation, epithelialization, and vascularization, impeding the repair and healing of the damaged urethra. More importantly, in response to pathological stress caused by factors such as urine and inflammation, urothelial fibroblasts differentiate into activated myofibroblasts that express α-smooth muscle actin (α-SMA), fibronectin (FN), and other matrix proteins, which are secreted into the extracellular matrix (ECM) [15]. Although activating myofibroblasts is considered a beneficial protective mechanism for urethral repair [16], the harsh urethral environment promotes the sustained overactivation of fibroblasts, resulting in excessive proliferation and deposition of collagen fibers and the formation of irreversible urethral scarring [[17], [18], [19]].

Smart responsive hydrogels, as an advanced biomaterial platform, offer a transformative solution to overcome the aforementioned challenges [[20], [21], [22]]. These hydrogels possess the capability to sense and respond to specific local microenvironmental cues—such as pH fluctuations, specific enzymes, ROS levels, temperature, or mechanical signals—enabling precise interventions, including the on-demand, spatially controlled, and temporally regulated release of therapeutic agents [[23], [24], [25]]. This characteristic is particularly crucial for the dynamic and complex urethral microenvironment, where conditions continuously evolve during the repair process. For instance, the early phase of urethral injury is often characterized by elevated ROS levels, while the passage of acidic urine induces significant pH alterations. In such scenarios, conventional single-intervention or static release strategies prove inadequate. Consequently, engineering hydrogels that utilize endogenous stimuli as triggering signals allows for the targeted delivery and localized intervention of therapeutics at the injury site. This approach maximizes therapeutic efficacy while simultaneously minimizing systemic exposure and off-target toxicities.

In current clinical practice, no effective drugs or bioactive molecules are approved for treating urethral injury or stricture. During our investigation into urethral scarring, we identified excessive fibrosis triggered by inflammatory stimuli as the primary pathological driver hindering urethral repair. While current anti-scarring drug research primarily targets skin, the distinct urethral microenvironment results in significantly more severe scarring compared to dermal wounds. Intriguingly, rheumatoid arthritis (RA), an inflammatory joint disorder, exhibits a pathological synovial microenvironment sharing key features with the injured urethra, including chronic moisture, sustained inflammation, and late-stage fibrotic transformation. This pathological parallelism suggests that RA-targeted therapeutics may offer novel strategies for urethral repair. Celastrol (Cel), a bioactive compound derived from the traditional Chinese herb *Tripterygium wilfordii*, has recently emerged as a promising RA therapeutic [26,27]. It demonstrates superior efficacy over conventional drugs like methotrexate, probably attributed to its multi-target anti-inflammatory mechanisms [28,29]. Critically, recent studies in cardiac, hepatic, and corneal fields indicate its potent anti-fibrotic activity via inhibition of fibroblast hyperactivation [[30], [31], [32]]. This compelling evidence strongly supports its therapeutic potential for urethral injury and stricture. However, its specific anti-inflammatory and anti-fibrotic effects within the urethral milieu, along with the underlying molecular mechanisms, remain unexplored. Furthermore, its clinical translation is hampered by poor water solubility and systemic toxicity [[33], [34], [35]]; consequently, conventional oral or intravenous administration fails to achieve localized therapeutic concentrations within the urethra while minimizing off-target effects, highlighting an urgent need for innovative drug delivery systems tailored to urethral repair.

Therefore, an ideal therapeutic strategy involves deploying an intelligent, stimuli-responsive hydrogel dressing. Such a platform would enable the controlled release of appropriate therapeutics to actively modulate the dynamic urethral microenvironment. This includes neutralizing ROS bursts and suppressing early-stage inflammation induced by injury or urine exposure, while simultaneously preventing persistent hyperactivation of urethral fibroblasts in later stages, ultimately facilitating scarless urethral reconstruction.

**Fig. 1:**
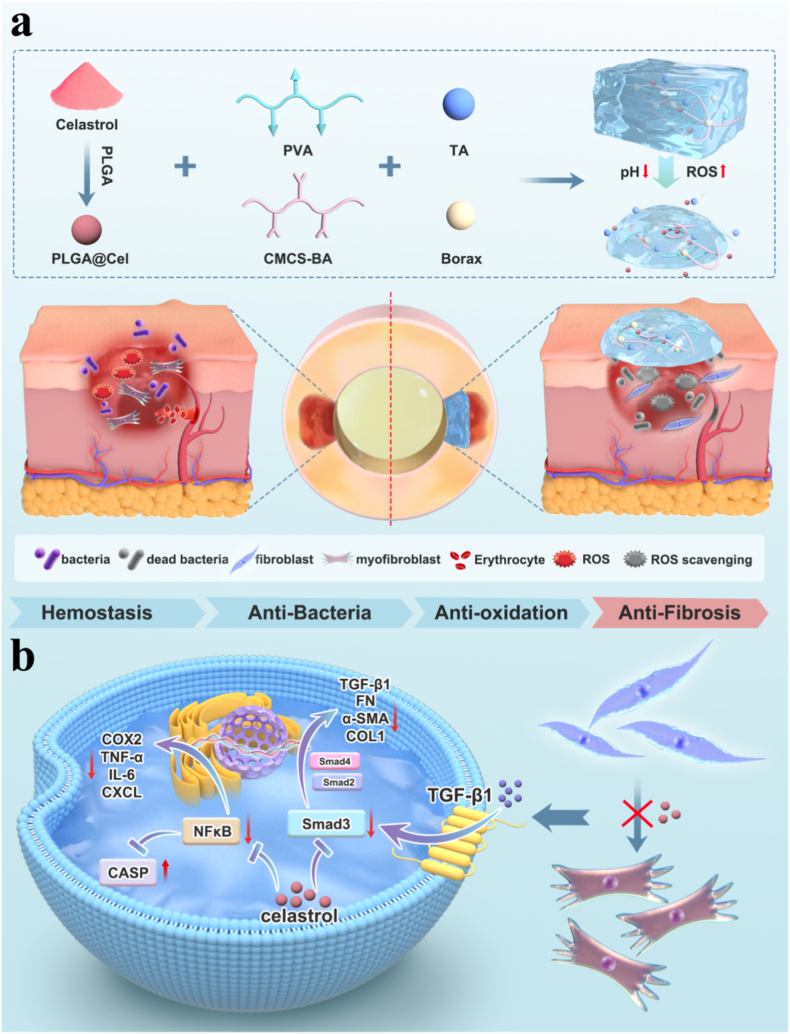
Schematic depiction of **a)** the fabrication of the CPT-Cel hydrogel and its application in treating urethral defects and **b)** an illustration detailing the role of celastrol in attenuating fibroblast activation.

Building upon this rationale, we designed and engineered a novel intelligent responsive hydrogel system. This system aims to efficiently encapsulate Cel and deliver it on demand with precision to the urethral injury site, enabling active modulation of the local microenvironment. The pH/ROS-responsive hydrogel base (denoted CP) was initially synthesized by forming dynamic boronate ester bonds between carboxymethyl chitosan-phenylboronic acid (CMCS-BA) polymer and poly (vinyl alcohol) (PVA) using borax as a crosslinker. Subsequently, tannic acid (TA), a natural polyphenol, was incorporated to endow the hydrogel with robust tissue adhesion, rapid hemostatic capability, and potent antibacterial properties, yielding the CPT hydrogel. Cel was encapsulated into poly (lactic-co-glycolic acid) (PLGA) nanoparticles (PLGA@Cel NPs), which were then uniformly integrated into the CPT hydrogel matrix, resulting in the multifunctional CPT-Cel hydrogel (Fig. 1). Upon application, the CPT-Cel dressing effectively adheres to and integrates with the urethral tissue, providing immediate hemostasis and antimicrobial defense. Critically, the hydrogel responds dynamically to the acidic pH and elevated ROS levels characteristic of the early injury phase. This responsiveness triggers the on-demand, controlled release of Cel, mitigating cellular damage caused by low pH and oxidative stress. Furthermore, sustained Cel release from the hydrogel matrix effectively inhibits the hyperactivation of urethral fibroblasts within the pro-fibrotic microenvironment, thereby preventing excessive collagen deposition during the scarring process. Comprehensive in vitro assays systematically validated the multifunctional efficacy of CPT-Cel, encompassing hemostasis, antibacterial activity, anti-inflammatory effects, and potent anti-fibrotic action. RNA sequencing elucidated the molecular mechanisms underlying Cel's anti-inflammatory effects and its suppression of fibroblast activation. In vivo evaluations based on histological and functional analyses demonstrated that CPT-Cel promotes early-stage re-epithelialization, angiogenesis, and cellular proliferation, while simultaneously reducing localized ECM deposition. This multifaceted approach restored urethral patency, achieving scarless healing and functional urethral reconstruction. This study not only effectively confirmed the translational value of Cel-based smart hydrogels in urethral repair but also proposed a dynamic microenvironment control strategy that provides a promising novel therapeutic avenue for the clinical management of urethral stricture.

## 2 Results and discussion

### 2.1 Preparation and characterization of hydrogels and nanoparticles

**Fig. 2:**
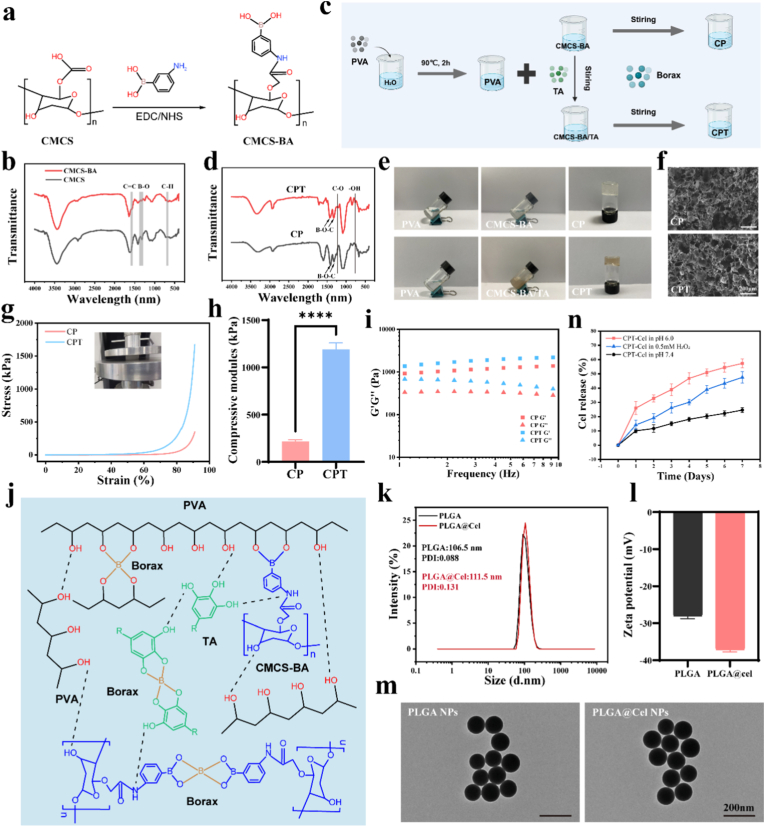
Synthesis and physicochemical analysis of hydrogels and nanoparticles. a) Preparation of CMCS-BA. **b)** FTIR spectrum of CMCS-BA. **c)** Preparation of CP and CPT hydrogels. **d)** FTIR spectra of CP and CPT. **e)** Precursor solutions and gelatinous forms of CP and CPT. **f)** SEM images of CP and CPT hydrogel cross sections. **g, h)** Stress–strain curves of the CP and CPT hydrogels (g) and their elastic modulus (h). **i)** Rheological properties of CP and CPT hydrogels. G″ and G′ represent the loss and storage modulus, respectively. **j)** Multiple complexation between CPT internal components. **k, l)** Size distribution (k) and Zeta potential (l) of PLGA and PLGA@Cel nanoparticles. **m)** TEM images of PLGA and PLGA@Cel nanoparticles. **n)** Dual-stimuli response and release curves of Cel from CPT-Cel in different solutions. Data are expressed as the mean ± standard deviation (SD) (n = 3). Two-tailed unpaired *t*-test was used to analyze statistical significance: ∗∗∗∗p < 0.0001.

Carboxymethyl chitosan (CMCS) is a chitosan derivative that is widely used in regenerative medicine [16,36,37]. 3-aminobenzene boric acid (BA) was grafted onto the side chain of CMCS in the presence of EDC/NHS (Fig. 2a). ^1^H NMR and FTIR were used to confirm whether the synthesized CMCS-BA polymers were successfully prepared. The ^1^H NMR spectrum of CMCS-BA (Fig. S1) showed a proton peak at 7.3–7.7 ppm corresponding to benzene rings, confirming that BA was successfully grafted onto the side chain of CMCS. In the FTIR spectrum of CMCS-BA (Fig. 2b), the vibrational peak in the range of 1500–1600 cm^−1^ was assigned to the C=C of benzene rings, and the vibrational peak at 1340 cm^−1^ was attributed to the B-O of boronic acid. In addition, the vibrational peak at 700 cm^−1^ was assigned to the stretching of the C–H bonds of the phenylboronic acid esters. The above results confirmed the successful preparation of CMCS-BA. Subsequently, CMCS-BA and PVA were rapidly crosslinked in the presence of borax to form a CP hydrogel with reversible, dynamic borate bonds (Fig. 2c–e). The peaks at 1424 and 1330 cm^−1^ were assigned to the asymmetric stretching of B-O-C, the peak at 1424 cm^−1^ was attributed to the tetrahedral complex, and the peak at 1330 cm^−1^ indicated the formation of a triangular complex (Fig. 2d). To confer adhesive and hemostatic properties to the CP hydrogel, TA was added to prepare the CPT hydrogel (Fig. 2c–e). The FTIR spectrum of the CPT hydrogel showed the characteristic absorption peaks of TA at 1204 and 754 cm^−1^, which were attributed to the stretching vibration of C-O of the ester moiety and the vibration of the planar -OH bond, respectively, confirming the successful preparation of the CPT hydrogel (Fig. 2d). Scanning electron microscopy (SEM) was used to observe the microstructures of the CP and CPT hydrogels, which revealed an ECM-like fibrous and porous microstructure (Fig. 2f). Furthermore, there was no significant difference in pore size between the two hydrogels (pore size of approximately 90 μm) (Fig. 2f), indicating that the addition of TA did not significantly affect the microstructure of the hydrogel. The elastic modulus and rheological properties of the two hydrogels were evaluated. CPT had a higher elastic modulus (Fig. 2g and h), energy storage modulus, and loss modulus than CP did (Fig. 2i), which indicated that TA effectively enhanced the mechanical properties of the hydrogel. This is attributed to the effective intermolecular hydrogen bonding network formed by the polyphenolic groups of TA in the hydrogel [[38], [39], [40]]. Energy dispersive spectroscopy (EDS) analysis and mapping were performed to determine the elemental distribution of the CPT hydrogel (Fig. S2). Multiple cross-linking between the internal components of the CPT hydrogel is shown in Fig. 2j. Preliminary testing of degradation rates revealed that the CPT hydrogel could degrade slowly in a simulated physiological environment, covering the urethral repair timeframe and meeting biosafety requirements (Fig. S3).

Owing to its hydrophobicity, Cel, when directly applied to the urethral lesion, may exhibit a short retention time and low bioavailability at the wound site, and thus Cel was encapsulated in PLGA NPs to improve its bioavailability and sustained release [34]. Dynamic light scattering (DLS) measurements showed that the size of PLGA NPs before and after drug loading were maintained at approximately 100 nm, and the polydispersity index (PDI) was less than 0.2, which indicated that the prepared NPs were well dispersed and homogeneous (Fig. 2k). Owing to the negative charge of Cel, the Zeta potential of PLGA NPs decreased from −28 mV to −37 mV after drug loading (Fig. 2l). Transmission electron microscopy (TEM) of the NPs (Fig. 2m) showed that the morphology of the NPs before and after drug loading was spherical and uniform in size. The standard curve obtained using UV–vis spectrophotometry revealed that the drug loading capacity of PLGA@Cel NPs was approximately 3.2% (Fig. S4).

It has been reported that excess Cel is cytotoxic, affecting the normal proliferation of cells [41,42]. To determine the optimal concentration for applying Cel, we therefore extracted and identified human urethral fibroblasts (HUFs) (Fig. S5) and treated them with Cel at different concentrations to determine cell viability via a CCK-8 assay. The results showed that HUF viability was unaffected by Cel at a maximum concentration of 0.1 μM (Fig. S6). Subsequently, we physically wrapped PLGA@Cel NPs with CPT to prepare a multifunctional smart hydrogel, denoted as CPT-Cel. SEM showed that the hydrogel microstructure was unchanged after the addition of drug-carrying nanospheres (Fig. S7). The degradation behavior of hydrogels under low pH or high ROS was shown in Fig. S8; the results demonstrated that the degradation of CPT-Cel was significantly slower in phosphate-buffered saline (PBS) (pH 7.4) than in an acidic environment (pH 6.0) or high-ROS environment (0.5 mM H_2_O_2_). Fig. 2n shows the release behavior of Cel in the hydrogel. After 1 day, the release amount in PBS was 10.09 ± 1.39%, and 27.30 ± 2.20% after 7 days, slightly lower than the degradation rate of hydrogels. The release of Cel was significantly accelerated in an acidic environment (pH 6.0) or a high-ROS environment (0.5 mM H_2_O_2_), which confirmed that the hydrogel responded to the dual stimuli of pH and ROS by releasing the encapsulated drug. Further testing of Cel release rates under PBS conditions revealed that it can sustain release for more than 28 days, meeting the requirements for urethral repair timeframe (Fig. S9). Thus, when the hydrogel is applied to the site of urethral injury, Cel can be released on demand in response to inflammatory ROS produced in the early stage of injury and acidic urine infiltration, while Cel will continue to be released as the hydrogel degrades in the later stage.

### 2.2 Mechanical and adhesive properties of CPT-Cel hydrogel

**Fig. 3:**
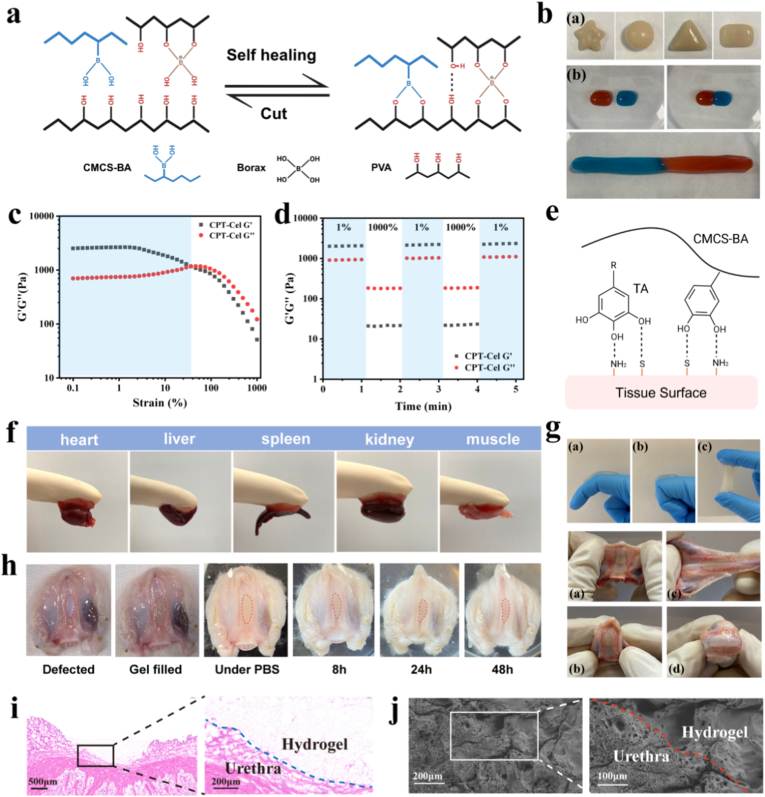
Self-healing and adhesive properties of the CPT-Cel hydrogel. **a)** Schematic of the self-healing properties of CPT-Cel. **b)** Visualization of hydrogel plasticity (a) and self-healing properties (b). **c)** G′ and G″ curves of CPT-Cel with strain increasing from 0.1% to 1000%. **d)** Self-healing of CPT-Cel varies with alternating changes between 1% and 1000%. **e)** Adhesive mechanism of CPT-Cel. **f)** Adhesion of CPT-Cel to different organs. **g)** Stretching and twisting of CPT-Cel after adhesion. **h)** Stability of CPT-Cel in flowing PBS **i, j)** H&E staining (i) and SEM (j) images of CPT-Cel after adhesion to the urethra.

The borate and phenyl borate bonds in CPT-Cel confer self-healing properties and excellent plasticity (Fig. 3a and b) [43]. Strain amplitude scanning of the CPT-Cel hydrogel showed that when the strain exceeded 40%, the storage modulus G′ intersected with the loss modulus G″, whereas when the strain exceeded 100%, the G′ of the hydrogel samples decreased rapidly, and the loss modulus was greater than the storage modulus (Fig. 3c). However, cyclic straining showed that the G' of hydrogel could rapidly recover to the original level when the strain reduced to 1% (Fig. 3d). This further confirmed that the hydrogel had good self-repairing properties, making it effective in sealing irregular urethral defects in real situations.

The presence of free phenylboronic acid in CPT-Cel, along with the catechol groups in TA, imparts a “cis-diol” functionality, mimicking the adhesive mechanism of catechol-based mussel adhesive proteins (Fig. 3e) [[44], [45], [46]]. This bio-inspired chemistry allows the CPT-Cel hydrogel to function as an effective urethral tissue sealant, while the hydration layer induced by urine prevents adhesion to the opposite urethral wall [[47], [48], [49]]. The CPT-Cel hydrogel immediately adhered to fresh organs upon contact, sustaining the weight of the tissue (Fig. 3f). Upon application, the hydrogel could rapidly fill and conformally seal complex urethral defects, forming a strong bond with the tissue matrix (Fig. 3g). This adhesive seal proved highly durable, remaining stable even when challenged by physiological fluid flow (simulated by flowing PBS) (Fig. 3g). Furthermore, the hydrogel demonstrated excellent mechanical compliance, adapting to the dynamic movements of underlying tissues. When subjected to significant stretching and twisting, the hydrogel-tissue interface remained intact without debonding or failure (Fig. 3h). Experiments demonstrated that shear forces from water flow could not dislodge the hydrogel, and the hydrogel could still effectively attach and seal the urethral wound after being impacted (Fig. S10). This characteristic is crucial for the hydrogel to adapt to the moist and dynamic environment of the urethra. Histological analysis through hematoxylin and eosin (H&E) staining and SEM clearly illustrated the formation of a tight, seamless interface between the CPT hydrogel and the urethra (Fig. 3i and j). The adhesive performance of the CPT hydrogel was quantitatively assessed using porcine skin shear testing, revealing a peak adhesive strength of 25.9 ± 2.9 kPa (Fig. S11), surpassing that of commercially available fibrin glue (approximately 10–20 kPa) [17,50,51]. These findings indicate that the hydrogel possesses the ideal adhesive and mechanical properties for a dynamic urethral wound dressing.

### 2.3 Biocompatibility and biological functions of CPT-Cel

**Fig. 4:**
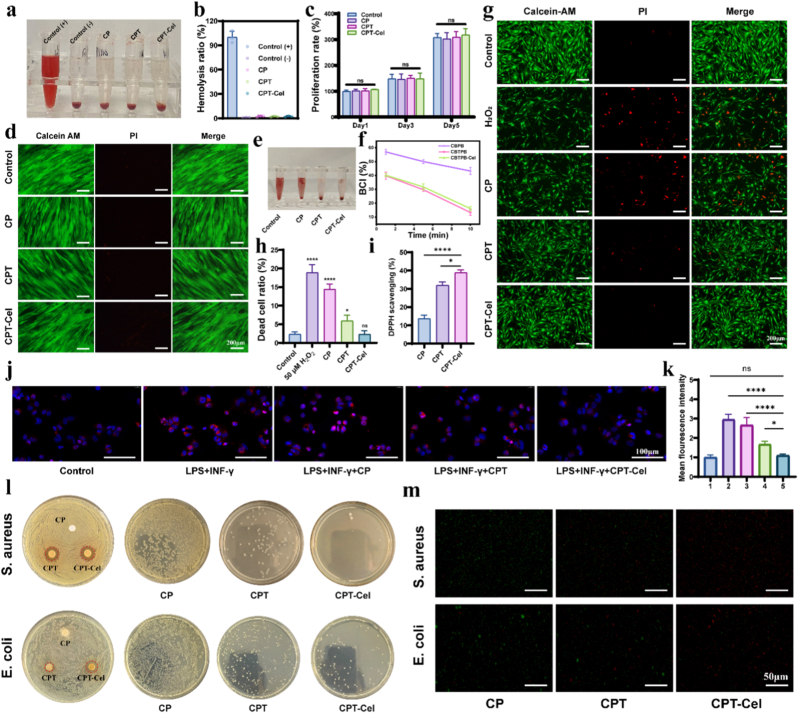
Biocompatibility and multifunctionality of hydrogels. a, b) Photographs of hemolysis experiments (a) and comparison of hemolysis rates (b). **c)** Cell proliferation after different treatments. **d)** Photographs of live/dead cell staining after different treatments. **e, f)** Photographs (e) and dynamic coagulation curves (f) at 10 min after different treatments. **g, h)** Live/dead staining of HUFs (g) and semi-quantitative analysis of dead cells (h) after treating HUFs for 24 h with H_2_O_2_ (50 μM) and different hydrogels. The asterisks indicate comparison with the Control. **i)** Comparison of DPPH clearance by the hydrogels. **j, k)** CD86 staining of macrophages after different treatments (j) and its semi-quantitative analysis (k). 1, 2, 3, 4 and 5 represent LPS + INF-γ, LPS + INF-γ+CP, LPS + INF-γ+CPT, and LPS + INF-γ+CPT-Cel, respectively. **l)** Antibacterial zone and colony count assays using *Escherichia coli* and *Staphylococcus aureus*. **m)** Bacterial live-dead staining with different hydrogel treatments. Data are expressed as the mean ± standard deviation (SD) (n = 3). One-way ANOVA followed by Dunnett's post hoc test was performed to analyze statistical significance: “ns” indicates no significance, ∗p < 0.05, ∗∗∗p < 0.001, ∗∗∗∗p < 0.0001.

Because the hydrogel might come into direct or indirect contact with blood, hemolysis testing was conducted. The results revealed that the hemolysis rates of all hydrogels were below the clinically acceptable threshold of 5% (Fig. 4a and b), indicating that the hydrogels exhibited excellent blood compatibility. Subsequently, the cytocompatibility of each hydrogel was assessed using HUFs. Cell viability assays conducted on days 1, 3, and 5 demonstrated that all hydrogels did not adversely affect cellular activity compared with the control (Fig. 4c). Additionally, live/dead cell staining assays indicated that the majority of cells treated with the hydrogels remained viable (green dots), with only negligible dead cells (red dots), indicating good cell growth (Fig. 4d). In addition, three-dimensional reconstruction performed after planting HUFs on CPT-Cel hydrogel confirmed the excellent biocompatibility of the hydrogel components (Fig. S12).

Rapid hemostasis following urethral injury is a fundamental requirement of biomaterials. We investigated the hemostatic effects of the various hydrogels using a blood clotting test to evaluate the blood clotting index (BCI). The dynamic coagulation curves (Fig. 4e and f) demonstrated that the hemostatic effect of CPT was significantly enhanced over that of CP. Although the BCI of CPT-Cel slightly decreased with time owing to the anticoagulation properties of Cel, it was still 16.1% after 10 min, which was similar to that of CPT (13.3%). These findings indicate that both the CPT and CPT-Cel fulfill the basic requirements for hemostasis in urethral wounds.

Inflammation in the early stage of urethral tissue injury or urine passage stimulates the excessive production of ROS, leading to cellular oxidative stress and death, which hampers the repair process. The ability of the hydrogel to protect urethral cells was assessed by constructing a cellular model of H_2_O_2_-induced oxidative stress in urethral fibroblasts [23]. The cytotoxic effects of different concentrations of H_2_O_2_ on urethral fibroblasts were examined by live/dead staining. As the concentration of H_2_O_2_ increased, the number of surviving HUFs (green color) decreased, indicating that the cytotoxic effect of H_2_O_2_ was concentration-dependent (Fig. S13). We then used H_2_O_2_ at a concentration of 50 μM for subsequent experiments. The extraction medium was obtained by soaking each hydrogel in cell culture medium for 24 h. The results revealed that 50 μM H_2_O_2_ induced significant cell death (18.9 ± 2.1%), while treatment with the hydrogels reduced the number of dead cells to varying degrees. Notably, CPT-Cel decreased the number of dead cells (2.3 ± 0.98%) to the level of that in the control group (2.37 ± 0.60%) (Fig. 4g and h). Therefore, the addition of TA and the loading of PLGA@Cel NPs effectively ameliorated oxidative damage to cells. Next, the antioxidative capacity of each hydrogel was evaluated by measuring the scavenging efficiency using a stabilizing radical, 2,2-diphenyl-1-picrylhydrazyl (DPPH). As shown in Fig. 4i, the DPPH scavenging efficiencies of CP, CPT, and CPT-Cel were 13.8%, 32.0%, and 38.9%, respectively. These results suggested that the hydrogels effectively scavenged exogenous ROS through their boron ester bonds, and the enhanced antioxidative capacity of CPT and CPT-Cel could be attributed to the excellent antioxidative activity of TA and Cel. Therefore, CPT-Cel loaded with PLGA@Cel NPs could effectively neutralize complex oxidative stress at the wound site on demand and protect wound-resident and regenerating cells.

In addition, we explored the immunomodulatory capacity of each hydrogel using macrophages. Lipopolysaccharide (LPS) and interferon-γ (IFN-γ) were added to the culture medium to mimic the inflammatory microenvironment in the early stage of urethral wounding [52]. The results showed that macrophage CD86 expression was significantly elevated in the inflammatory environment, indicating its conversion to M1 (pro-inflammatory type), and after the addition of each hydrogel, CD86 expression decreased to different degrees, with CPT-Cel decreasing CD86 expression to the lowest level (Fig. 4j and k). This further corroborates the robust immunomodulatory capacity of CPT-Cel, highlighting its potential in mitigating the exacerbated inflammatory response in the early stage of urethral injuries.

The antimicrobial properties of the hydrogels are important for preventing urethral wound infections. The antimicrobial effects of the hydrogels were preliminarily assessed with a bacterial inhibition ring test using *E. coli* and *Staphylococcus aureus*, revealing that CPT and CPT-Cel maintained a more pronounced inhibitory ring than CP (Fig. 4l). Then, the bacterial suspensions were co-cultured with each hydrogel, and after 24 h, they were subjected to live/dead staining and uniformly applied to bacterial culture plates. The bactericidal effect of the hydrogel gradually increased with the addition of TA and Cel, and the CPT-Cel exhibit the most potent bactericidal effect (Fig. 4l and m). Given that biofilm formation is a major challenge in urinary tract infections, we further evaluated the anti-biofilm capability of the hydrogels using a crystal violet biofilm assay. The results demonstrated that the CPT-Cel hydrogel significantly inhibited the biomass of established biofilms for both bacterial strains compared to other groups (Fig. S14). Collectively, these findings confirm that the CPT-Cel hydrogel possesses comprehensive antibacterial properties, meeting the clinical requirements for protecting urethral wounds.

### 2.4 Cel attenuates HUFs overactivation in vitro by disrupting TGF-β and NF-κB pathways

**Fig. 5:**
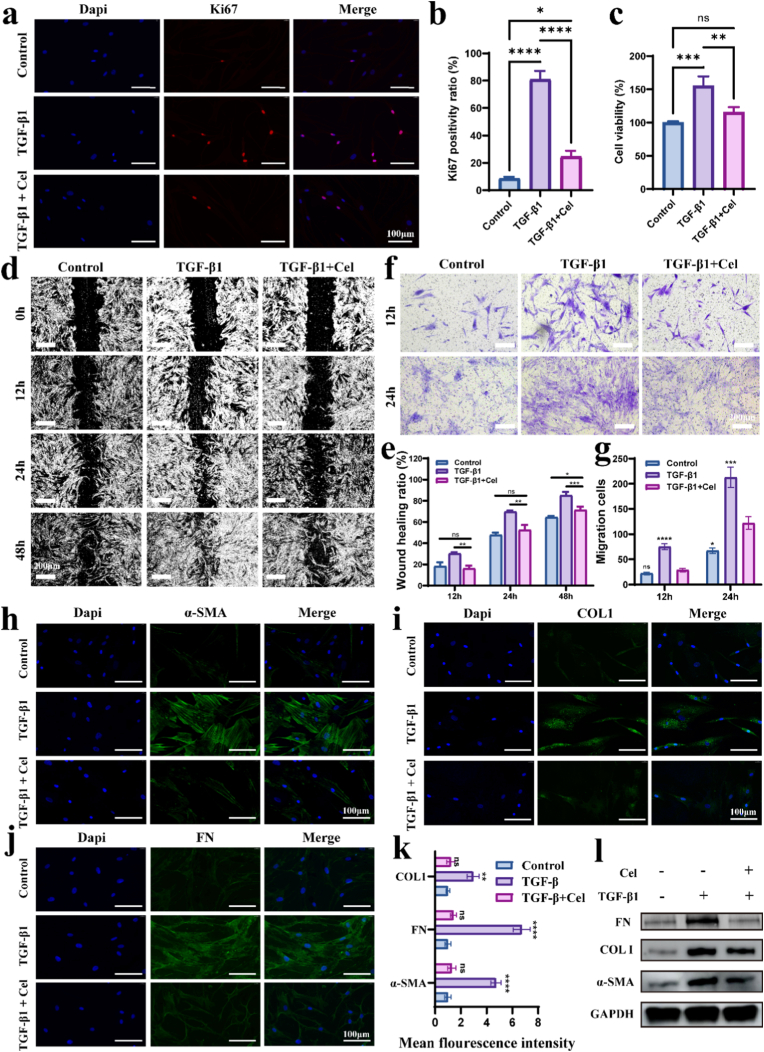
Cel inhibits HUFs overactivation. a, b) Representative images of Ki67 and DAPI co-staining of fibroblasts (a) and its semi-quantitative analysis (b). **c)** Results of CCK8 after different treatments. **d, e)** Microscopic examination of different groups during cell scratch tests (d) and statistical analysis (e). (scale bar = 200 μm). **f, g)** Microscopic examination of different groups during cell Transwell tests (f) and statistical analysis (g). The asterisks indicate comparison with the TGF-β1+Cel group. (scale bar = 200 μm). **h–k)** Immunofluorescence staining of α-SMA, COL1, and FN in fibroblasts after different treatments (h–j) and their semi-quantitative analysis (k). The asterisks indicate comparison with the TGF-β1+Cel group. **i)** WB visualization of α-SMA, COL1, and FN in fibroblasts after different treatments. Data are expressed as the mean ± standard deviation (SD) (n = 3). One-way ANOVA followed by Tukey's or Dunnett's post hoc test was performed to analyze statistical significance: “ns” indicates no significance, ∗*p* < 0.05, ∗∗*p* < 0.01, ∗∗∗*p* < 0.001, ∗∗∗∗*p* < 0.0001.

Urethral fibroblasts are the main cell type in urethral tissue and play a central role in scar formation after urethral injury [6,10]. We isolated and identified human urethral fibroblasts (HUFs) from human urethral tissue (Fig. S5) to validate the anti-fibrotic effect of Cel. TGF-β1, as a classic pro-fibrotic factor, is used in vitro to simulate pro-fibrotic stimuli. We treated HUFs with 15 ng/mL TGF-β1 for 48 h to activate their differentiation into myofibroblasts. The inhibitory effect of Cel (0.1 μM) on the proliferation of activated HUFs was evaluated via Ki67 fluorescence staining. Co-incubation with Cel significantly reduced the Ki67-positive rate of activated myofibroblasts, which approached the level of the control group (Fig. 5a and b). CCK-8 assays of different experimental groups also indicated that Cel treatment inhibited the proliferation of activated HUFs, with a statistically significant difference (Fig. 5c). Scratch and Transwell assays were performed to observe whether Cel could inhibit TGF-β1-induced HUF migration. The results of the scratch assay showed that the migration rate of cells treated with TGF-β1 reached 85.14 ± 2.61% after 48 h, while that of activated cells subsequently treated with Cel decreased to 71.40 ± 2.43% (Fig. 5d and e). Transwell migration assays of different groups showed that Cel treatment significantly inhibited the migration of TGF-β1-induced HUFs. After 24 h, the number of migrated cells in the control group, the TGF-β1 group, and the TGF-β1 + Cel group was 74.3 ± 7.8, 212.7 ± 20.1, and 113.0 ± 9.2, respectively (Fig. 5f and g). The above results indicated that the activation of HUFs into myofibroblasts was significantly inhibited by Cel.

Consistently, immunofluorescence staining and Western blotting (WB) showed that TGF-β1 activated HUFs by significantly upregulating fibrotic markers, such as α-SMA, collagen І (COLІ), and FN, whereas this up-regulation was significantly inhibited by Cel (Fig. 5h–l). In addition, immunofluorescence staining showed that Cel directly inhibited the expressions of TGF-β1 and TGF-βRІІ induced by TGF-β1 (Fig. S15), suggesting that TGF-β pathway may be one of the pathways through which it inhibits fibroblast activation. However, its specific mechanism of inactivation requires further investigation.

**Fig. 6:**
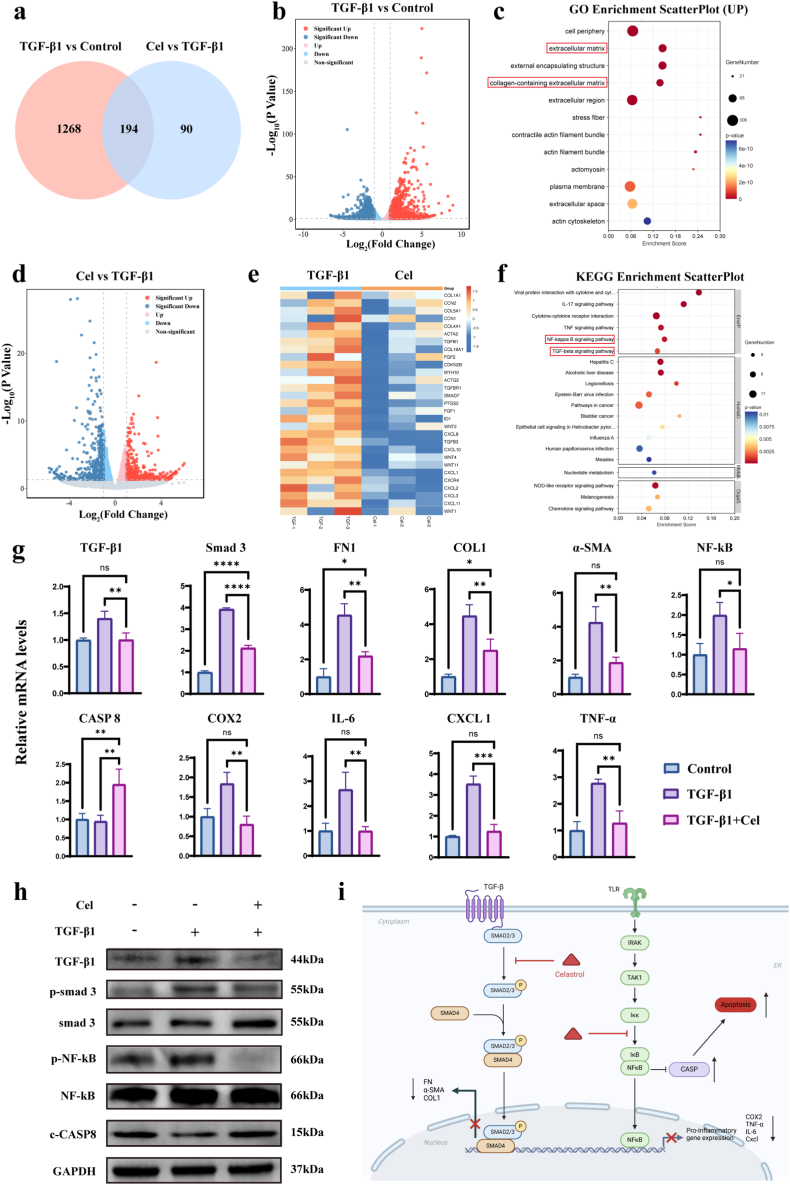
Exploration of the mechanism by which Cel inhibits HUFs overactivation. a) Venn diagram of differentially expressed genes (DEG) of TGF-β1 vs Control and Cel vs TGF-β1. **b**) DEG volcano diagram of TGF-β1 vs Control. **c**) GO analysis of TGF-β1 vs Control. **d**) DEG volcano diagram of Cel vs TGF-β1. **e**) Differences in fibrosis and scarring related genes between Cel vs TGF-β1. **f**) KEGG analysis of Cel vs TGF-β1. **g, h**) Relative mRNA levels of related genes from real-time quantitative PCR (g) and expression levels of key proteins from WB (h). **i**) Schematic representation of the mechanism by which Cel inhibits fibroblast overactivation. Data are expressed as the mean ± standard deviation (SD) (n = 3). One-way ANOVA followed by Dunnett's post hoc test was performed to analyze statistical significance: “ns”indicates no significance, ∗p < 0.05, ∗∗p < 0.01, ∗∗∗p < 0.001, ∗∗∗∗p < 0.0001.

We performed RNA sequencing to identify differentially expressed genes (DEGs) in HUFs. Using the thresholds of |log_2_FC|≥1 and false discovery rate (FDR) q < 0.05, a large number of DEGs were found among the three groups (Fig. 6a, S16). “TGF-β1 vs Control” representing the number of DEGs after TGF-β1 treatment compared with the control group was 1462 (807 + 655) DEGs (Fig. 6b), which was attributed to the activation of the TGF-β pathway by TGF-β1. Moreover, Gene Ontology (GO) analysis showed that TGF-β1 treatment significantly upregulated DEGs related to the regulation of the ECM and collagen-containing ECM (Fig. 6c). However, the effect of TGF-β1 was significantly alleviated after co-treatment with Cel, which effectively downregulated fibrosis-related genes (Fig. 6d and e). Furthermore, Kyoto Encyclopedia of Genes and Genomes (KEGG) analysis indicated that DEGs were significantly enriched in signaling pathways of cytokine–cytokine receptor interaction, NF-κB, and TGF-β (Fig. 6f). Subsequently, qPCR analysis was carried out on the key genes in these pathways (Fig. 6g). We found that Cel significantly inhibited the TGF-β signaling pathway in myofibroblasts, evidenced by reduced transcription of TGF-β1 and Smad3, which consequently led to the suppression of downstream fibrotic effector molecules, including α-SMA, COL1, and FN. In addition, NF-κB, a key protein in the NF-κB signaling pathway was significantly downregulated, reducing the transcription levels of pro-inflammatory factors such as COX2, IL-6, IL-8, and CXCL1. Interestingly, the downregulation of NF-κB signaling pathway was accompanied by the upregulation of cysteine-dependent aspartate-specific Protease-8 (CASP8), indicating increased level of apoptosis,which was confirmed by apoptosis assays (Fig. S17). Determining the expression levels of key proteins in two pathways yielded consistent results (Fig. 6h). This series of changes, including the direct inhibition of the TGF-β pathway, downregulation of pro-inflammatory and pro-fibrotic molecules in the NF-κB pathway, and promotion of apoptosis, ultimately attenuated the activation of fibroblasts (Fig. 6i). Therefore, Cel can serve as an effective drug to curb urethral wound fibrosis and subsequent excessive scar hyperplasia.

### 2.5 CPT-Cel promotes urethral repair and reduces scarring

**Fig. 7:**
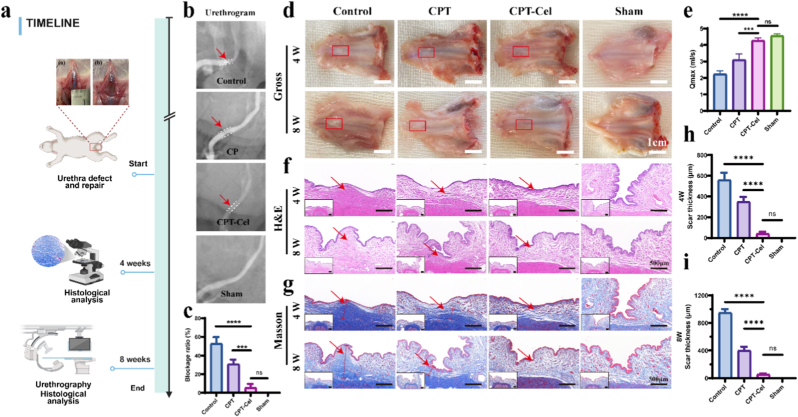
In vivo functional and histological analysis. a) Timeline of in vivo urethral reconstruction. **b)** Representative images of urethrography of each group. Red arrows show the strictured segments. **c)** Comparison of urethral blockage rate of each group at 8 weeks. **d)** Photographs of the gross morphology. Red boxes represent areas of injury. **e)** Comparison of maximal urinary flow rate of each group at 8 weeks. **f, g**) Histological images of tissues stained with H&E (e) and Masson trichrome (f) at 4 and 8 weeks. **h, i)** Scar thickness of each group at 4 (g) and 8 weeks (h). One-way ANOVA followed by Dunnett's post hoc test was performed to analyze statistical significance: “ns” indicates no significance, ∗∗∗p < 0.001, ∗∗∗∗p < 0.0001.

The in vivo biological effects of the CPT-Cel hydrogel dressing were investigated using a rabbit model of urethral injury. As shown in Fig. 7a and S18, after creating a urethral defect of a certain size, an appropriate amount of hydrogel was applied, which completely covered and adhered to the urethral defect. At 8 weeks post-operation, urethrography was used to evaluate the degree of urethral obstruction in each group (Fig. 7b and c). The Control group showed a very narrow lumen owing to excessive scar formation, with an obstruction rate of 52.6 ± 7.0%. The CPT group showed a relatively wider urethral lumen, while the CPT-Cel group exhibited a smooth lumen similar to that of the sham-operated group, with an obstruction rate of only 5.6 ± 3.9%. Gross morphology (Fig. 7d) showed that in the Control group, scar formation occurred at 4 weeks and accelerated at 8 weeks, representing the worst repair effect. The CPT group had reduced scarring, although visible scars remained. Urethral repair in the CPT-Cel group was significantly better than that in the other groups. The repaired urethra was smooth, with no obvious hypertrophic scar formation. In addition, the maximum urine flow rate (Qmax) further confirmed that the CPT-Cel group exhibited the best treatment effect, with Qmax reaching 4.27 ± 0.15 mL/s, which was not significantly different from that of the sham-operated group (4.56 ± 0.12 mL/s) (Fig. 7e). Urethral tissues were stained with H&E, Masson trichrome, and Sirius red to histologically evaluate urethral scarring and collagen deposition (Fig. 7f–g, S19). Compared with the Control group (558.3 ± 58.2 μm) and CPT group (349.0 ± 38.0 μm), the CPT-Cel group had the thinnest scar (40.7 ± 14.8 μm) at 4 weeks (Fig. 7h). The gap widened at 8 weeks, with the scar thicknesses of the Control group, CPT group, and CPT-Cel group were 950.7 ± 41.3 μm, 399.0 ± 45.0 μm, and 54.7 ± 15.0 μm, respectively (Fig. 7i). Sirius red staining showed that the CPT-Cel group had the least deposition of type I collagen among the treatment groups (Fig. S19). These results indicated that the CPT-Cel hydrogel successfully inhibited the formation of urethral scars and the occurrence of urethral strictures after urethral injury. No significant implant reactions or infections were observed during tissue harvesting, and rabbits remained in good health, indicating that all scaffolds are biocompatible. Following 8 weeks of CPT-Cel scaffold application, rabbit major organs (heart, lungs, liver, spleen, and kidneys) underwent H&E staining to assess any histopathological alterations (Fig. S20). Compared to the normal group, no significant lesions or abnormalities were observed in organs from rabbits treated with the CPT-Cel scaffold, indicating no systemic toxicity associated with PLGA@Cel or Cel was detected under experimental conditions.

**Fig. 8:**
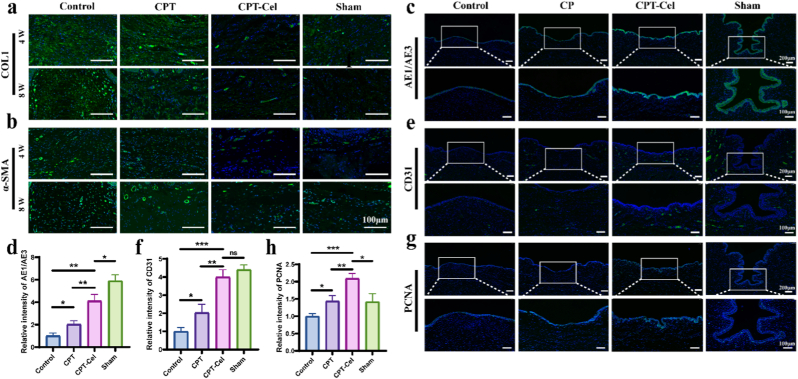
In vivo immunofluorescence staining. a, b) Immunofluorescence staining of COL1 (a) and α-SMA (b) was performed on the 4/8-week groups to examine extracellular matrix levels**. c–h**) Immunofluorescence staining of AE1/AE3 (c, d), CD31 (e, f), and PCNA (g, h) in tissues to examine epithelial formation, angiogenesis, and cellular proliferation at 4 weeks. Data are expressed as the mean ± standard deviation (SD) (n = 3). One-way ANOVA followed by Tukey's post hoc test was performed to analyze statistical significance: “ns” indicates no significance, ∗p < 0.05, ∗∗p < 0.01, ∗∗∗p < 0.001.

Multiple approaches were employed to further evaluate the in vivo repair process and outcomes. Immunofluorescence staining of α-SMA and COL1 showed that the control group and CPT group secreted excessive extracellular matrix, while the extracellular matrix levels in the CPT-Cel group and sham surgery group tended to be similar (Fig. 8a and b). This pattern was consistent with the extent of scar formation and urethral narrowing observed in each group. In addition, we examined macrophage polarization and inflammatory cytokine levels during the acute phase of urethral injury (day 3 post-operation). Immunofluorescence staining for the M1 marker CD86 and the M2 marker CD206, together with analysis of inflammatory cytokines in tissue homogenates, showed that treatment with the CPT-Cel hydrogel markedly increased the M2/M1 macrophage ratio and reduced the levels of pro-inflammatory cytokines IL-1β, IL-6, and TNF-α in urethral tissue (Fig. S21), demonstrating a pronounced immunomodulatory effect. More importantly, during the intermediate phase of repair (4 weeks post-operation), the expression of key mediators in the TGF-β and NF-κB pathways, including TGF-β1 and NF-κB, was significantly decreased in the CPT-Cel group (Fig. S22), confirming effective in vivo inhibition of fibrogenic signaling by Celastrol, in line with our in vitro findings. Rapid epithelization, vascularization, and cell proliferation early in the tissue repair process are crucial to avoid excessive scarring [16]. Immunofluorescence staining was used to evaluate urethral epithelialization (AE1/AE3), vascularization (CD31), and cell proliferation (PCNA) in each group. The results showed that at 4 weeks, the levels of epithelialization, angiogenesis, and cell proliferation in the control group were significantly lower than those of the other groups. This could be attributed to the excessive production of ROS in the early stage and the erosive effect of acidic urine, which hindered wound repair. In the CPT-Cel group, on-demand release of TA and Cel in the early stage of injury prevented persistent inflammation, which protected cells and promoted epithelial hyperplasia, blood-vessel growth, and cell proliferation, accelerating the repair process (Fig. 8c–h). At the end point of repair (8 weeks), there was no significant difference in the number of epithelial layers and proliferating cells among the groups. However, the CPT-Cel group and the sham-operated group had the largest number of blood vessels (Fig. S23). These results indicated that the CPT-Cel hydrogel promoted the early restoration of the normal urethral structure, which was beneficial for the normal function of the urethra and, to some extent, improved the outcome of urethral scarring.

As previously described, the hostile dynamic urethral microenvironment following injury—characterized by bacterial contamination from urine, an acidic wound milieu, and excessive ROS—constitutes a primary impediment to early wound repair and a key driver of subsequent scarring. To address this, we applied celastrol, a compound possessing multi-target anti-inflammatory and anti-fibrotic properties, to urethral repair. Furthermore, to overcome the significant challenges of Cel's poor aqueous solubility and systemic toxicity, we innovatively engineered a pH/ROS dual-responsive intelligent hydrogel system (CPT-Cel). Owing to its reliable adhesion and plasticity, the CPT-Cel hydrogel dressing we designed can rapidly fix and completely cover urethral defects, initially isolating external stimuli. In addition, it has beneficial hemostatic and antibacterial properties, which promote early blood coagulation at the wound site and prevent common infections by *E. coli* and *S. aureus*. More importantly, the hydrogel responds to the excessive level of ROS in the urethral wound and the decrease in pH from urine passage by immediately degrading via their dynamic borate ester bonds/phenyl borate bonds to release Cel, which provides anti-ROS, anti-inflammatory, and antibacterial effects. This timely removal of excess ROS prevents cell damage, reduces the aggregation of inflammatory cells, and accelerates the repair process. Therefore, this precise treatment system releases the therapeutic drug, Cel, on demand, which not only improves the treatment outcome but also reduces the drug-administration frequency and drug side-effects. In the middle and late stages of injury repair, overactivation of fibroblasts is the main challenge of urethral scar healing, which is closely related to persistent inflammation and the harsh urine environment (such as urine infiltration). At this time, the CPT-Cel hydrogel dressing slowly releases Cel as the hydrogel degrades, attenuating fibroblast activation. Specifically, Cel can directly inhibit the migration and proliferation of fibroblasts in a profibrotic environment. Inhibiting the TGF-β pathway also inhibits the transcription of effector molecules such as α-SMA, Col1, and FN, which restricts ECM secretion and collagen deposition. In addition, Cel can disrupt the NF-κB signaling pathway, reduce the expression level of pro-inflammatory and pro-fibrotic factors such as COX2, IL-6, IL-8, and CXCL1, and at the same time increase the level of apoptosis, weakening the overactivation of fibroblasts and effectively preventing and curbing scar hyperplasia caused by urethral fibroblasts. Therefore, the CPT-Cel hydrogel dressing we designed can actively regulate the unique urethral microenvironment, effectively exerting hemostatic, antibacterial, anti-inflammatory, and antifibrotic effects during the urethral repair process, ultimately achieving scar-free reconstruction of urethral injuries.

Notably, this study also has limitations. Our research was carried out using a healthy rabbit model of urethral injury, which may not effectively simulate the real-life clinical situations of patients with urethral injuries (complications or underlying diseases such as infection and diabetes). In addition, because many patients already have urethral scars that affect urination, the effect of the hydrogel dressing after scar resection needs further exploration. In the future, we will apply this hydrogel dressing to larger animal models (such as minipigs or canine models) utilizing chronic post-injury or inflammatory urethral stricture models. This will further broaden the applicability of the hydrogel dressing by addressing translational challenges related to replicating the complex pathology of human urethral stricture, providing clinically relevant urethral dimensions and surgical approaches, and enabling long-term assessment of hydrogel efficacy, integration, and safety, ultimately promoting its clinical translation. We believe that the on-demand strategy for treating urethral injuries proposed in this work, combined with the anti-inflammatory and antifibrotic effects of Cel, has broad application prospects.

## 3 Conclusion

This study aims to address the challenge of scarless urethral reconstruction through active modulation of the urethral microenvironment. The engineered CPT-Cel hydrogel dressing exhibits remarkable adaptability to this microenvironment, effectively maintaining its dynamic homeostasis. Possessing multifunctional properties, the dressing provides robust tissue adhesion, rapid hemostasis, potent antibacterial activity, anti-inflammatory effects, and effective anti-fibrotic action. These attributes enable the dressing to accelerate the repair process while concurrently inhibiting hyperplastic events during scarring, ultimately achieving functional scarless reconstruction of the injured urethra. Beyond validating the therapeutic efficacy of Cel delivered via an intelligent hydrogel platform and elucidating its core mechanisms in modulating the dynamic urethral microenvironment, the proposed strategy offers a promising new paradigm for the clinical translation of urethral biomaterials.

## 4 Materials and methods

*Materials:* Carboxymethyl chitosan (CMCS), N-hydroxysuccinimide (NHS), 1-ethyl-3-(3-dimethylaminopropyl) carbodiimide hydrochloride (EDC·HCl), 3-aminobenzene boric acid (BA), polyvinyl alcohol (PVA), tannic acid (TA), borax, and celastrol (Cel) were purchased from Aladdin Biochemical Technology Co. DPPH assay kit was purchased from Elabscience Biotechnology Co.,Ltd. Crystal violet staining solution were acquired from Servicebio. Calcein-AM/PI Assay Kit (U23-002A) was purchased from YOBlBlO (Shanghai, China). lipopolysaccharide (LPS), phorbol-12-myristate-13-acetate (PMA), and interferon-γ (IFN-γ)—were obtained from Beyotime Biotechnology Co. Ltd. The THP-1 monocytic cell line was procured from the National Collection of Authenticated Cell Cultures (Chinese Academy of Sciences). Cryopreservation solution (New Cell & Molecular Biotech, C40100) was used for the cryopreservation of these cells.

*Characterization:* Morphological analysis was performed using scanning electron microscopy (SEM; ZEISS Sigma 300). Chemical structure of CMCS-BA polymer was determined by 1H nuclear magnetic resonance spectroscopy (^1^H NMR; Bruker Avance II, 400 MHz). Functional groups were identified through Fourier-transform infrared spectroscopy (FTIR; Nicolet iS20). Elemental composition was analyzed by energy-dispersive X-ray spectroscopy (EDS; Oxford Ultim Max 100). Rheological properties were characterized using a rotational rheometer (HAAKE MARS 60). Compressive strength and adhesive properties were assessed with a universal testing machine (INSTRON 5542). In this work, the compressive modulus of the samples was calculated as the slope of the linearly fitted data (typically around 60-80% of max stress), which was the same across all groups. Cell behaviors were monitored using laser scanning confocal microscopy (Leica SP5) and fluorescence microscopy (Olympus IX70-S1F2).

*Synthesis of phenylboronic acid-grafted carboxymethyl chitosan (CMCS-BA):* CMCS was dissolved in deionized water to configure a 1 wt% CMCS solution of 500 mL. BA (6.0 g), EDC·HCl (4.0 g), and NHS (1.5 g) were then added sequentially and stirred thoroughly for 48 h. After dialysis and lyophilization, CMCS-BA was obtained.

*Preparation and characterization of hydrogels:* PVA was completely dissolved in water with stirring at 90 °C for 2 h to obtain a 7 wt% PVA solution. The concentration of CMCS-BA solution was 1 wt%; 2% TA was added to 1 wt% CMCS-BA solution to obtain CMCS-BA/TA (CT) solution. Subsequently the CMCS-BA solution or CT solution was mixed 1:1 with the PVA solution, followed by the addition of 1% Borax and stirring to obtain CMCS-BA/PVA(CP) or CT/PVA (CPT) hydrogels. For the loading of PLGA@Cel microspheres, they were directly mixed with PVA solution in advance, and CPT-Cel hydrogel was obtained after the above steps.

*Preparation and characterization of PLGA@Cel nanoparticles:* The dispersed phase was prepared by dissolving 100 mg of PLGA and 10 mg of Cel in 20 mL of chloroform and 3 mL of acetone, respectively, and homogeneous ultrasonication after co-mixing; the continuous phase was prepared by adding a certain amount of SDS and PVA into 500 mL of deionized water, and dissolving by stirring thoroughly in a water bath with heat (60 °C).The dispersed phase was added into the reservoir, and the monodisperse O/W emulsion was obtained by pressing the dispersed phase into the continuous phase through the SPG microporous membrane under the applied pressure of 0.06 MPa. The O/W emulsion was transferred to a thermostatic bath at 40 °C and stirred at 500 r/min for 6∼8 h. With the evaporation of organic solvents chloroform and acetone, the PLGA@Cel microspheres were finally cured. The particle size distribution and zeta potential of PLGA@Cel nanoparticles (NPs) were determined using dynamic light scattering (DLS, Malvern Zetasizer). The morphology of PLGA@Cel NPs was observed under transmission electron microscopy (TEM). The mass of loaded Cel was detected and calculated using a UV-visible spectrophotometer based on the standard curve of Cel (425 nm). The loading efficiency (LE%) of Cel was determined by the following equation: LE (%) = (mass of loaded Cel)/(mass of loaded Cel + mass of PLGA in the nanoparticles) × 100%.

*Degradation ratio:* To evaluate degradation profiles, hydrogels were weighed (initial weight, w0) and incubated in: (i) PBS (pH 7.4), (ii) PBS (pH 6.0), or (iii) PBS (pH 7.4) containing 0.5 mM H_2_O_2_. At designated time points, samples were retrieved, blotted dry, and weighed (final weight, wt). Degradation ratio (Δw) was calculated using: Δw = (w0 - wt)/w0 × 100%.

*Harvest and culturing of HUFs:* Human urethral fibroblasts (HUFs) were isolated from fresh surgical specimens obtained during urethroplasty procedures. Tissues were rinsed three times with PBS, minced, and digested with 0.1% collagenase I (Sigma-Aldrich) at 37 °C. Digestion was terminated by adding complete medium, followed by filtration (100 μm mesh) (KGN1405-1, KeygenBioTECH) and centrifugation (300×*g*, 5 min). Cell pellets were resuspended in DMEM (Dakewe, 66016111) supplemented with 10% FBS (Cyagen, FBSSR-01021) and 1% penicillin/streptomycin maintained under standard culture conditions. The human relevant experiments were approved by the Ethics Committee of Shanghai Sixth Peoples Hospital (2020-141).

*In vitro drug release:* Cel release kinetics from CPT-Cel hydrogel (MWCO: 50 kDa) were assessed in: (i) PBS (pH 7.4), (ii) PBS (pH 6.0), or (iii) PBS (pH 7.4) containing 0.5 mM H_2_O_2_. At predetermined intervals, aliquots were collected, and Cel concentration was quantified using UV-Vis spectrophotometry at 425 nm.

*Adhesion tests and adhesion interface analysis:* To evaluate the tissue-adhesive properties of the hydrogel, an ex vivo adhesion test was performed. Rats were euthanized via an intraperitoneal overdose of pentobarbital sodium, in accordance with the approved animal ethics protocol. Major organs and tissues, including the heart, liver, kidney, spleen, and muscle, were immediately harvested. The hydrogel's ability to adhere to the surfaces of these fresh tissues was then assessed qualitatively. Hydrogel adhesion strength was tested using lap shear tests, where CPT-Cel was placed between two pieces of pig skin and fixed for 1 min, and subsequently clamped in a universal testing machine and tensile loaded at a strain rate of 10 mm/s. The hydrogel adhesion strength was determined at the separation point. The hydrogel was adhered to the rabbit urethra and then dehydrated by free drying or frozen section, and the interface was examined by SEM or stained by H&E to observe the interfacial integration between the hydrogel and the urethra.

*Hemolysis Experiment:* Fresh anticoagulated blood was collected from the marginal ear vein of healthy adult New Zealand rabbits. Blood samples (0.5 mL) were mixed with: (i) 10 mL distilled water (positive control), (ii) 10 mL PBS (negative control), (iii) 10 mL PBS + 0.1 g CP, (iv) 10 mL PBS + 0.1 g CPT, or (v) 10 mL PBS + 0.1 g CPT-Cel. After thorough mixing and 60 min incubation at 37 °C, samples were centrifuged (116×*g*, 10 min). Supernatant absorbance was measured at 540 nm using a microplate reader. Hemolysis rate was calculated as: [(Asample - Anegative)/(Apositive - Anegative)] × 100%.

*Cell viability:* For drug toxicity assessment, cells were treated with Cel at graded concentrations (0.01, 0.1, 1, and 10 μM) for 24 h. For hydrogel toxicity testing, extract media were prepared by immersing hydrogels (CP, CPT, CPT-Cel) in culture medium (1 g/10 mL) for 24 h. Cell proliferation and viability were assessed after 1, 3, and 5 days of exposure. In anti-fibrosis studies, experimental groups received TGF-β1 (15 μg/mL) or TGF-β1 + 0.1 μM Cel, while control groups received untreated medium. After 48 h treatment, 10 μL CCK-8 (B34302, Selleckchem.com) was added per well, followed by 2 h incubation at 37 °C. Absorbance was measured at 450 nm using a microplate reader.

*BCI Experiment:* Hydrogel samples (0.1 g each of CP, CPT, and CPT-Cel) were placed in centrifuge tubes. Anticoagulated whole blood (100 μL, containing 10 μM CaCl_2_) was applied onto each hydrogel and incubated at 37 °C. At designated time points (1, 5, 10 min), 1 mL deionized water was added, followed by vortexing and centrifugation (116×*g*, 10 min). Supernatant absorbance (A1) was measured at 540 nm. For total hemoglobin determination, 100 μL blood (containing 10 μM CaCl_2_) was mixed with 1 mL deionized water, and absorbance (A2) was measured. BCI was calculated as A1/A2, with time-dependent BCI curves generated.

*Live/dead assay:* Fibroblast viability was evaluated after 3-day culture in hydrogel extracts using live/dead staining. Briefly, cells were incubated with dual fluorescent dyes: Petri dishes (CellPro Biotechnology, 803100B) were rinsed three times with PBS and then treated with Calcein-AM (2 μM) and propidium iodide (PI, 4 μM) for 20 min under light-protected conditions. Fluorescence images were obtained using a fluorescence microscope. CPT-Cel hydrogel was freeze-dried for 24h in advance and exposed to UV light for 24 h. HUFs were digested with trypsin (0.25%) and then collected by centrifugation. HUFs were prepared as a cell suspension at a concentration of 1.0 × 10^6^. By immersing the CPT-Cel into the cell suspension, HUFs was fully absorbed and evenly distributed within the hydrogel. HUFs were directly cultured within CPT-Cel hydrogel for 72 h, followed by live/dead staining and 3D image reconstruction to visualize cell spatial distribution.

To establish an oxidative stress model, fibroblasts were exposed to H_2_O_2_ at graded concentrations (10, 50, 100 μM) for 24 h at 37 °C. For protective effect evaluation, cells were pre-treated with hydrogel extracts prior to challenge with 50 μM H_2_O_2_ for 24 h. Cell viability was determined through calcein-AM/PI staining, as described above, with quantitative analysis performed using ImageJ software to calculate live/dead cell percentages.

*DPPH scavenging effect:* Hydrogel extracts (5 μL) from experimental groups were mixed with 195 μL of DPPH working solution in 96-well plates. After 30 min dark incubation, absorbance was measured at 515 nm using a microplate reader, with PBS serving as blank control. DPPH scavenging activity was calculated as: [(OD_sample_ - OD_Blank_)/OD_Blank_] × 100%.

*Hydrogel effect on macrophage polarization:* THP-1 monocytes were differentiated into M0 macrophages via 24 h treatment with 200 ng/mL PMA. M1 polarization was induced using complete medium containing 100 ng/mL LPS and 20 ng/mL IFN-γ. Experimental groups were treated with hydrogel extracts supplemented with LPS/IFN-γ, while control groups received LPS/IFN-γ in extract-free medium. Following 36 h culture in 6-well plates, M1 marker expression (CD86) was assessed through immunofluorescence staining, as described below.

*Antibacterial test:* Antimicrobial activity assessment was performed using two complementary methods. For the inhibition zone assay, sterile paper discs impregnated with hydrogel extracts were placed on agar plates pre-coated with bacterial suspensions (10^6^ CFU mL-1) and incubated at 37 °C for 24 h. Zone diameters were measured from digital images of inhibition halos. For the colony counting assay, bacterial suspensions (10^6^ CFU mL-1) were co-cultured with hydrogel samples for 24 h prior to plating on fresh agar media. Following additional 24 h incubation at 37 °C, bacterial colonies were observed.

*Immunofluorescence Analysis:* Immunofluorescence analysis was conducted to identify cellular markers in HUFs (Vimentin) and markers in urethral samples (CD31, PCNA, AE1/AE3, α-SMA, COL1, TGF-β1, NF-kB, CD86, CD206), along with proliferation (Ki-67), fibrotic (α-SMA, COL1, FN), and TGF-β pathway components (TGF-β1, TGF-βR1) in HUFs. For immunostaining, tissue sections were blocked with 3% BSA prior to overnight incubation at 4 °C with primary antibodies in a humidified chamber. Following three 5-min PBS washes (pH 7.4), sections were incubated with fluorophore-conjugated secondary antibodies for 1 h at room temperature under light-protected conditions. Nuclear counterstaining was performed using DAPI (4′,6-diamidino-2-phenylindole) for 5 min, followed by final PBS washes. Fluorescence imaging was carried out using an epifluorescence microscope, with semi-quantitative analysis performed via Image J software.

*Cell migration:* Wound healing assays were performed by creating linear scratches in confluent fibroblast monolayers using 1000 μL pipette tips guided by a sterile ruler. Wound closure was monitored at specified intervals using phase-contrast microscopy, with quantitative analysis of cell-free areas conducted via Image J software. For migration assays, HUF suspensions were seeded in the upper chambers of 24-well Transwell plates (725301, NEST Biotechnology) containing serum-free DMEM, while the lower chambers contained: (i) complete medium, (ii) complete medium supplemented with 15 μg/ml TGF-β1, or (iii) complete medium with 15 μg/ml TGF-β1 plus Cel (0.1 μM). Following 12/24 h incubation, cells were stained with crystal violet for quantitative assessment under bright-field microscopy.

*RNA sequencing:* RNA isolation was conducted across treatment groups employing TRIzol™ reagent (Life Technologies). Functional annotation analysis was performed through Kyoto Encyclopedia of Genes and Genomes (KEGG) pathway enrichment and Gene Ontology (GO) classification, utilizing bioinformatics platforms provided by OE Biotech.

*qRT-PCR tests:* Total RNA extraction was performed from treated fibroblasts across experimental groups using TRIzol reagent (Life Technologies). First-strand cDNA synthesis was conducted and quantitative real-time PCR was carried out on a QuantStudio™ system using SYBR Green Pro Taq HS qPCR Kit II (Rox Plus) (ACCURATE BIOTECHNOLOGY (HUNAN) CO., LTD, ChangSha, China (AG11719)). Gene-specific primers for NF-κB, COX2, IL-6, IL-8, CXCL1, CASP8, TGFB1, Smad3, α-SMA, COL1A1, and fibronectin (FN), along with GAPDH (endogenous control), are detailed in Table S1. Relative mRNA expression levels were normalized to GAPDH and calculated via the comparative CT method (2– ΔΔCT) (n = 3).

*Western blot:* Cellular lysates were prepared from experimental groups using RIPA buffer supplemented with protease/phosphatase inhibitors. Following complete lysis on ice, samples were centrifuged to isolate supernatants. Determine the protein concentration of the sample using the BCA Protein Concentration Assay Kit (Cat No.20201ES76; Yeasen, Shanghai, China). Protein aliquots (20 μg/lane) were resolved on 10% SDS-PAGE and electrophoretically transferred to PVDF (0.45 μm) membranes. Membranes were blocked with Rapid Protein Free Closure Solution (BR0051-01, ACE, China) for 1 h at 37 °C prior to overnight incubation at 4 °C with primary antibodies specific for: NF-kB (phosphorylated/native), CASP8, TGFB1, Smad3 (phosphorylated/native), α-SMA, COL1, FN, and GAPDH (loading control). Post-incubation, three TBST washes preceded 1 h incubation with HRP-conjugated secondary antibodies. Protein signals were visualized using chemiluminescence imaging systems.

*HUFs apoptosis assay:* An Annexin V-FITC apoptosis kit was utilized to evaluate the apoptotic effect of celastrol on activated HUFs. HUFs were seeded in 6-well plates at a density of 5 × 10^5^ cells per well. Following a 12-h attachment period, the cells were exposed to various treatment media for 24 h. Subsequently, the HUFs were harvested and stained according to the kit protocol. Apoptosis rates were then determined by flow cytometry.

*In vivo urethral defect and reconstruction:* All animal procedures were approved by the Experimental Animal Ethics Committee of Shanghai Sixth People's Hospital (Approval No. 2025-0054) and were performed in strict accordance with the ARRIVE (Animal Research: Reporting of In Vivo Experiments) guidelines. A total of 36 male New Zealand white rabbits (2.0-2.5 kg) were housed in a facility under a 12-h light/dark cycle, at a controlled temperature of 22 ± 2 °C, with ad libitum access to standard chow and water. The sample size was chosen based on previous well-established studies on rabbit urethral reconstruction, which demonstrated that a sample of n = 3 per group at each time point is sufficient for robust histological and morphological evaluation, while adhering to the 3Rs principle (Replacement, Reduction, and Refinement) for this exploratory study. The animals were randomly assigned to four groups (n = 9 per group): Control (defect only), CPT hydrogel, CPT-Cel hydrogel, and Sham-operated. Animals from each group were euthanized at 3 days, 4 or 8 weeks post-surgery (n = 3 per group at each time point) for evaluation. Anesthesia was maintained as needed throughout the procedure. For surgery, animals were anesthetized with an intravenous injection of pentobarbital sodium through the marginal ear vein. Urethral injury modeling was performed in all groups, except for the sham-operated group, in which the urethra was not injured during surgery. After mucosal defects (0.5 × 0.8 cm) were created by scissors, different hydrogels were immediately applied to fill the mucosal defects in the corresponding groups, while the control group received no treatment. After surgery, animals were catheterized for three days, and monitored individually to prevent injury.

*Maximum Flow Rate (Qmax) and urethrography:* Prior to euthanasia at Week 8, rabbits underwent urethral cystography and urinary flow rate assessment. During Qmax testing, animals were handled gently in a quiet environment to minimize stress. After anesthesia, a sterile 8F catheter was inserted, followed by infusion of 150–200 mL sterile saline. The catheter was subsequently removed, and the rabbit was allowed to urinate spontaneously upon regaining consciousness. The Qmax was recorded using a urine flow rate detector during this process. This procedure aimed to evaluate urinary function under conditions simulating natural voiding. During urethrography, an 8F catheter was inserted into the urethra, followed by radiographic imaging post-injection of iodine contrast. Urethral stenosis severity was quantified by calculating the stenosis-to-total urethral width ratio across experimental groups.

*Euthanasia and Tissue Harvesting:* At the scheduled endpoints (3 days, 4 or 8 weeks), animals were humanely euthanized via an intravenous overdose of pentobarbital sodium administered through the marginal ear vein. Following confirmation of death, urethral specimens were harvested. IL-1*β*, IL-6, and TNF-*α* cytokines in the tissue homogenates of each group at 3 days were quantified using ELISA kits according to the manufacturer's instructions. Tissue processing involved 24-h immersion in 4% neutral buffered formalin, proceeding through dehydration to paraffin embedding. Serial sections (4 μm) were obtained for histopathological evaluation using hematoxylin-eosin (H&E), Masson's trichrome, and Sirius red staining protocols. 8 weeks post-treatment, major organs (heart, liver, spleen, lungs, kidneys) were collected from rabbits in the normal and CPT-Cel groups for further HE staining.

*Statistical analysis:* Data are presented as mean ± standard deviation (SD). Statistical differences between two groups were analyzed using two-tailed *t*-test. For comparisons among more than two groups, one-way ANOVA followed by Tukey's post hoc test was performed. A p-value <0.05 was considered statistically significant, using asterisks to denote significance levels: ∗P < 0.05, ∗∗P < 0.01, ∗∗∗P < 0.001, and ∗∗P < 0.0001. These analyses were conducted using SPSS 25.0 (SPSS Inc., USA).

## CRediT authorship contribution statement

**Yangwang Jin:** Conceptualization, Data curation, Methodology, Writing – original draft. **Fei Qin:** Data curation, Investigation, Software. **Ranxing Yang:** Data curation, Formal analysis, Methodology. **Wenzhuo Fang:** Data curation, Formal analysis, Methodology. **Kaile Zhang:** Data curation, Investigation. **Meng Liu:** Investigation, Resources. **Ming Yang:** Formal analysis, Investigation, Supervision, Visualization. **Ying Wang:** Supervision, Visualization, Writing – review & editing. **Qiang Fu:** Funding acquisition, Supervision, Writing – review & editing.

## Declaration of competing interest

The authors declare that they have no known competing financial interests or personal relationships that could have appeared to influence the work reported in this paper.

## Data Availability

Data will be made available on request.
