# Supplementary material for: Dynamic microenvironment-regulated hydrogels releasing celastrol lead to urethral scarless repair
Source: Mater Today Bio. 2026 Feb 11;37:102917. doi: 10.1016/j.mtbio.2026.102917 (PMC12926572; doi:10.1016/j.mtbio.2026.102917)
Supplement: Multimedia component 1 [file mmc1.docx]

Supplementary material for

**Dynamic microenvironment regulated hydrogels releasing celastrol lead to urethral scarless repair**

Yangwang Jin^1#^, Fei Qin^2#^, Ranxing Yang^1#^, Wenzhuo Fang^1#^, Kaile Zhang^1^, Meng Liu^1^, Yuhui Wang^1^, Ming Yang^1^* , Ying Wang^1^*, Qiang Fu^1^*

^1^Department of Urology, Shanghai Sixth People’s Hospital Affiliated to Shanghai Jiao Tong University School of Medicine, Shanghai Eastern Institute of Urologic Reconstruction, Shanghai Jiao Tong University, Shanghai 200233, China.

^2^Department of Urology, Wuxi 9th People's Hospital, 999 Liangxi Road, Binhu District, Wuxi 214061, Jiangsu Province, China
*Corresponding author E-mail: jamesqfu@126.com (Q. Fu), sdzbbswangying@alumni.sjtu.edu.cn (Y. Wang), yangminguro@163.com (M. Yang)

# These authors contributed equally.

This file includes:

**Figs. S1 to S23**

**Table S1**


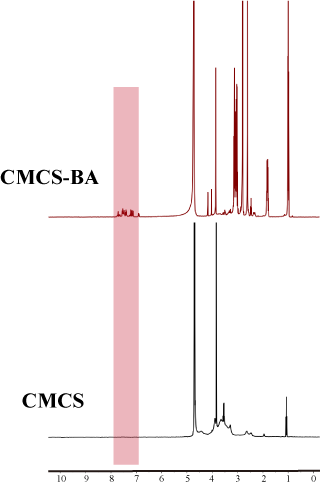


**Fig. S1.** ^1^HNMR spectra of CMCS-BA polymer.


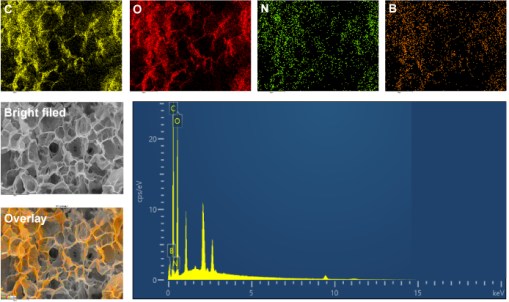


**Fig. S2.** EDS spectrum and mapping images of the CPT hydrogel.


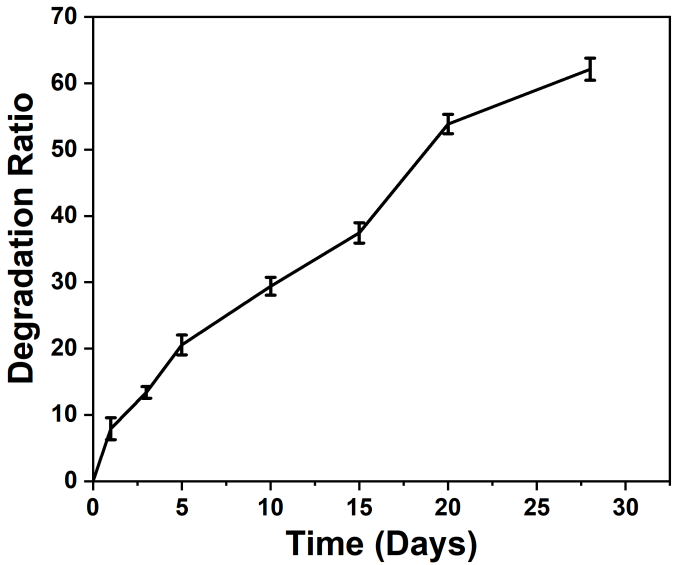


**Fig. S3.** Degradation ratio curve of CPT hydrogel in PBS 7.4.


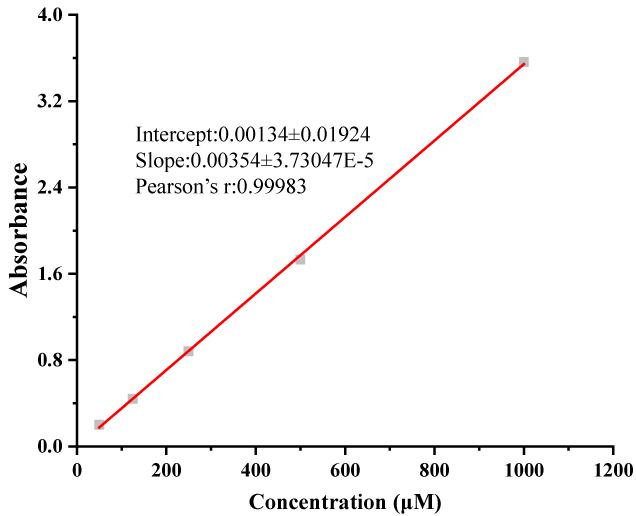


**Fig. S4.** Cel concentration standard curve at 425 nm.


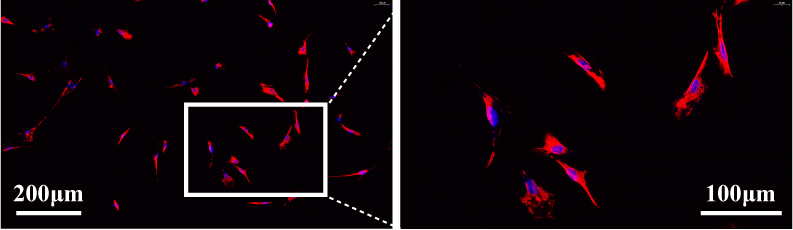


**Fig. S5.** Representative images of HUFs co-stained with Vimentin and DAPI.


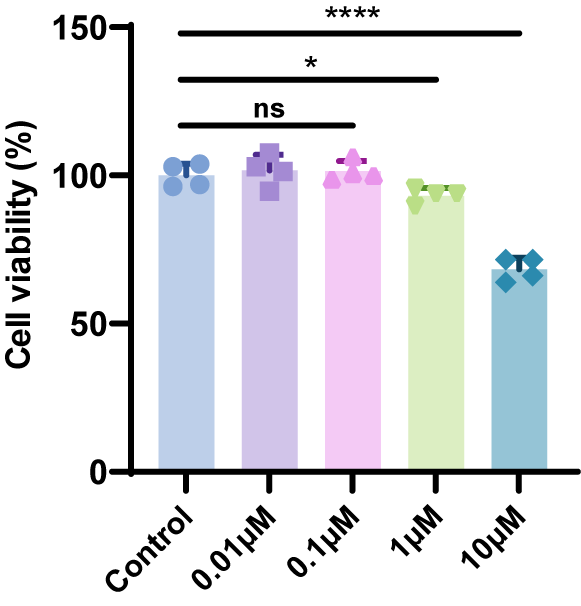


**Fig. S6.** Cytotoxicity testing of different concentrations of Cel. One-way ANOVA followed by Dunnett's post hoc test was performed to analyze statistical significance: “ns” indicates no significance, *p < 0.05, ****p < 0.0001.


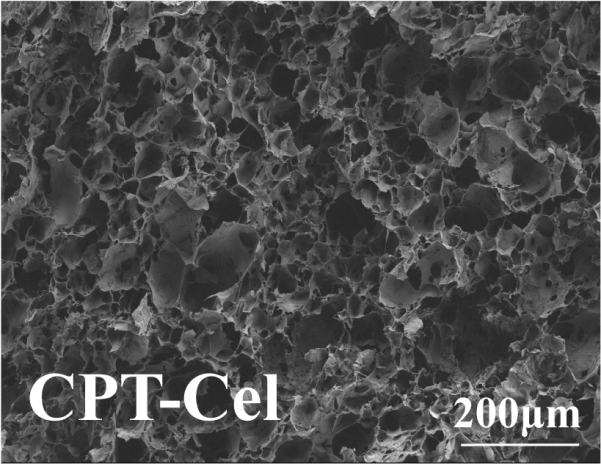


**Fig. S7.** Scanning electron microscope (SEM) image of CPT-Cel hydrogel.


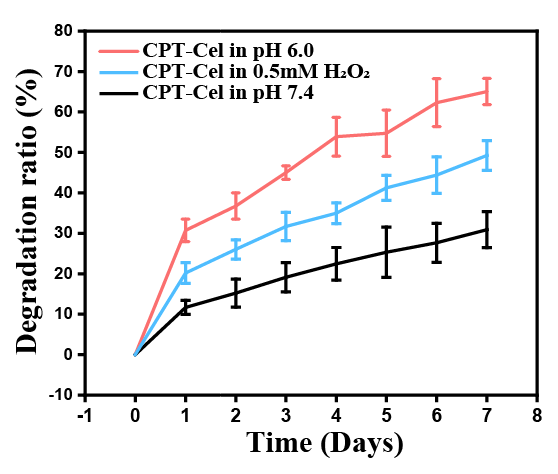


**Fig. S8.** Dual-stimuli response and degradation curves of CPT-Cel in different solutions.


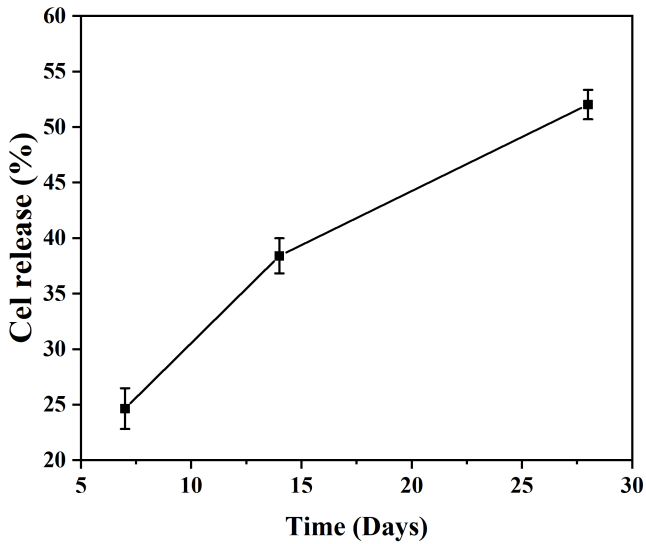


**Fig. S9.** Cel release curve in PBS 7.4.


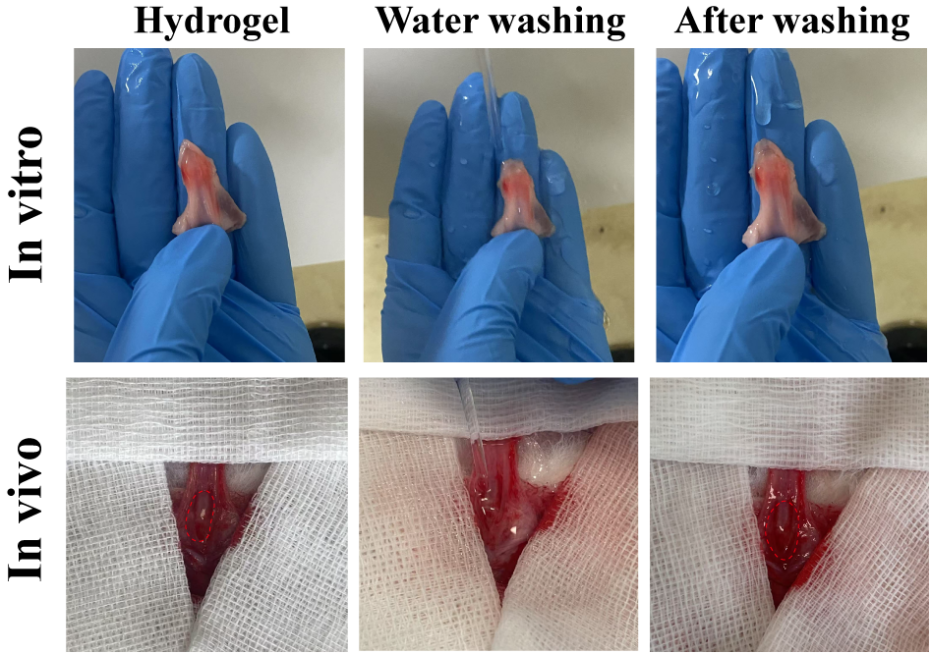


**Fig. S10.** Representative photographs showing the resistance to water washing at approximately 10 kPa water pressure. Red dashed boxs represent the adhesive hydrogels.


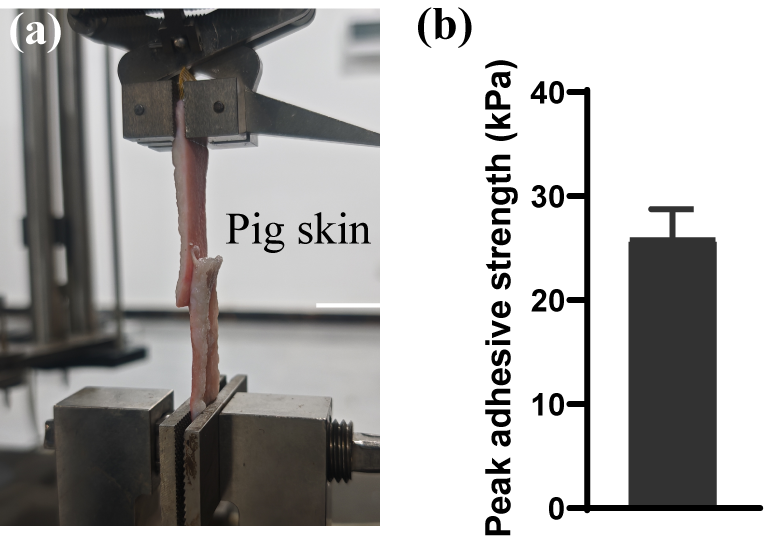


**Fig. S11.** Procedure (a) and results (b) of pig skin adhesion experiments.


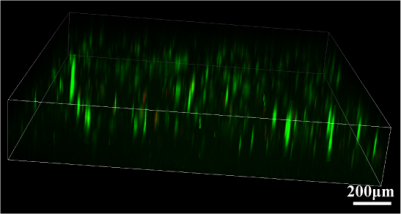


**Fig. S12.** Live-dead stained HUFs in the CPT-Cel hydrogel (scale bar = 200 μm).


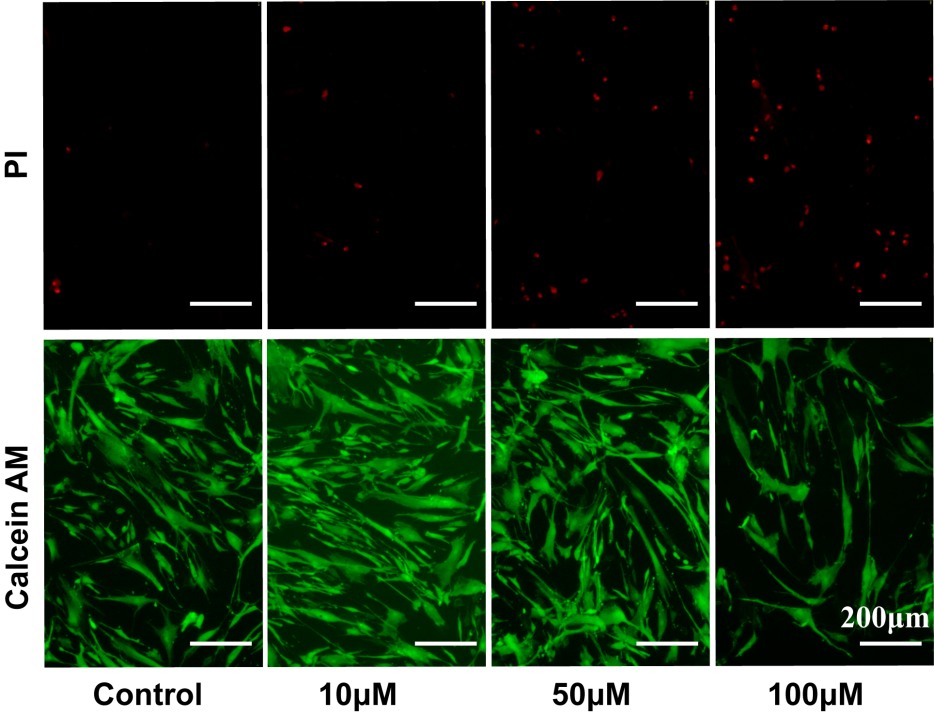


**Fig. S13.** Live-dead stained HUFs after treatment with different concentrations of H_2_O_2_.


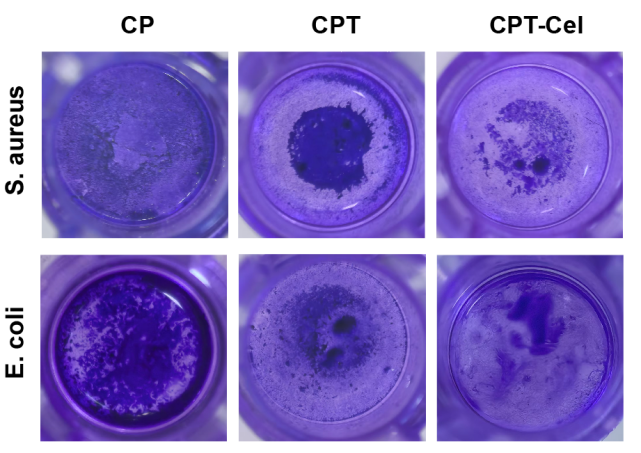


**Fig. S14.** Images of *Escherichia coli* and *Staphylococcus aureus* biofilm stained with crystalline violet.


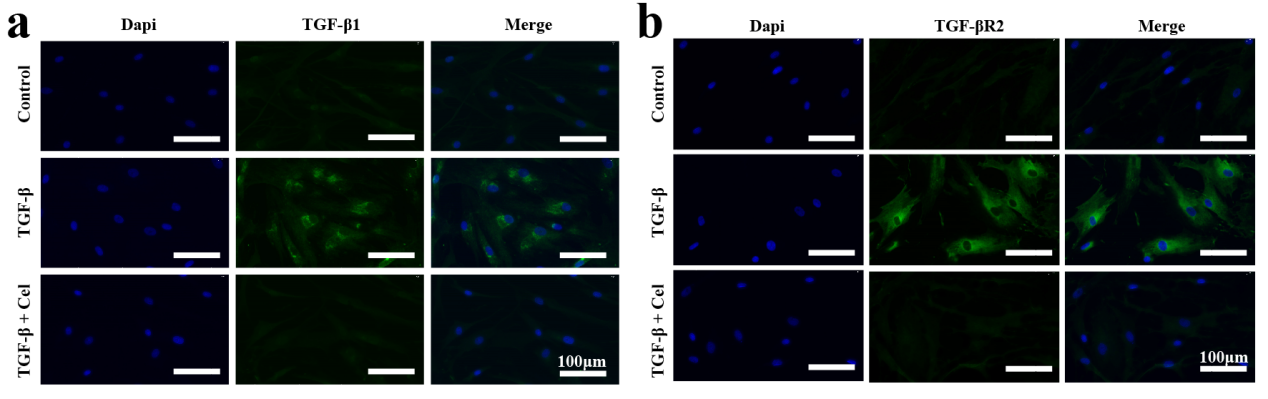


**Fig. S15.** a) Representative images of HUFs co-stained with TGF-β1 and DAPI. b) Representative images of HUFs co-stained with TGF-βR2 and DAPI.


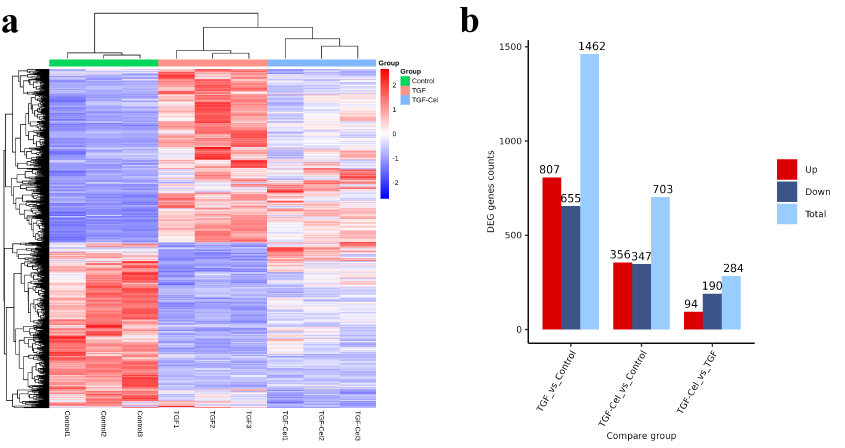


**Fig. S16.** a) Heat map analysis of samples among 3 groups. b) Number and distribution of DEGs among 3 groups.


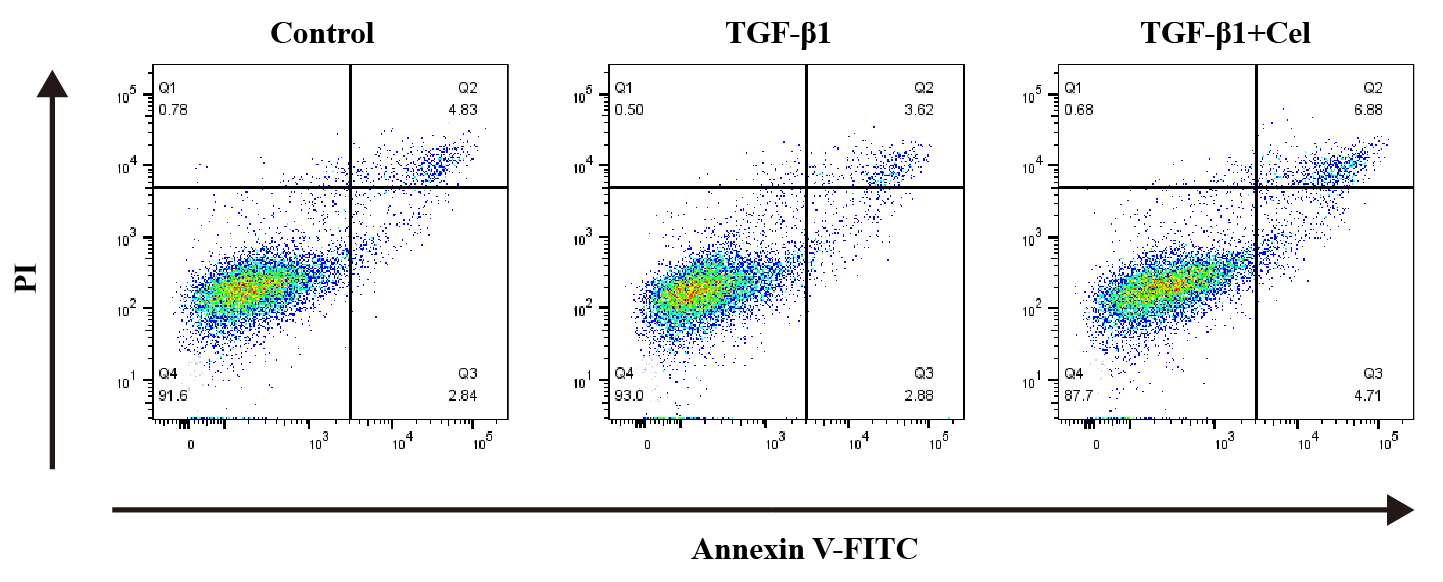


**Fig. S17.** Flow cytometric results of AnnexinV-FITC/PI double-staining assay of HUFs.


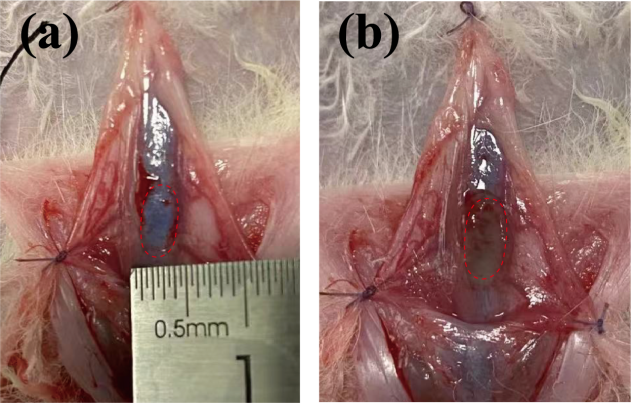


**Fig. S18.** Urethral injury and repair surgical procedure. a) urethral injury. b) Hydrogel filling and adhering to the urethral injury.


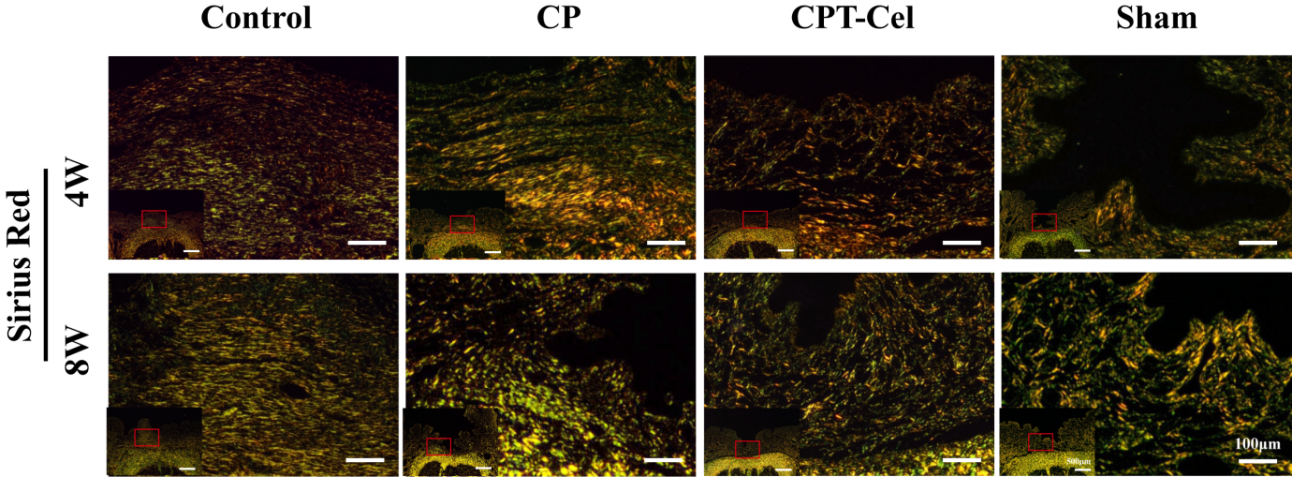


**Fig. S19.** Sirius red stained images of the urethra in each group after 4 and 8 weeks.


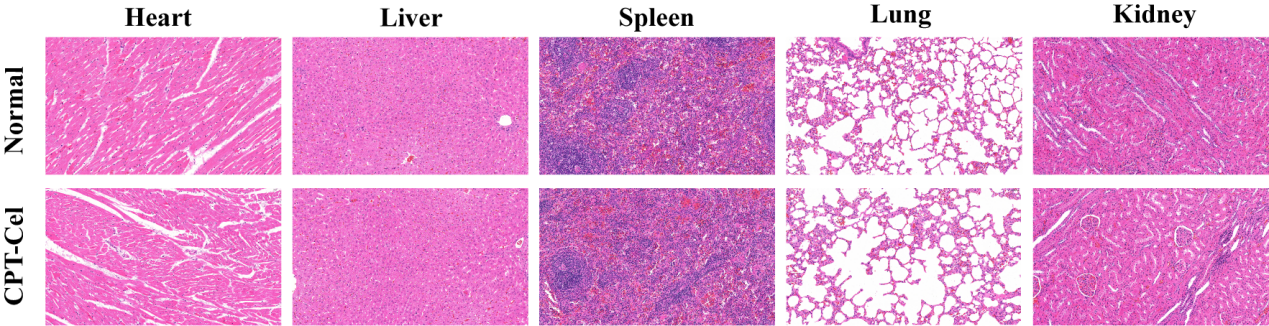


**Fig. S20.** Histopathological examination of the rabbit’s major organs 8 weeks after treatment.


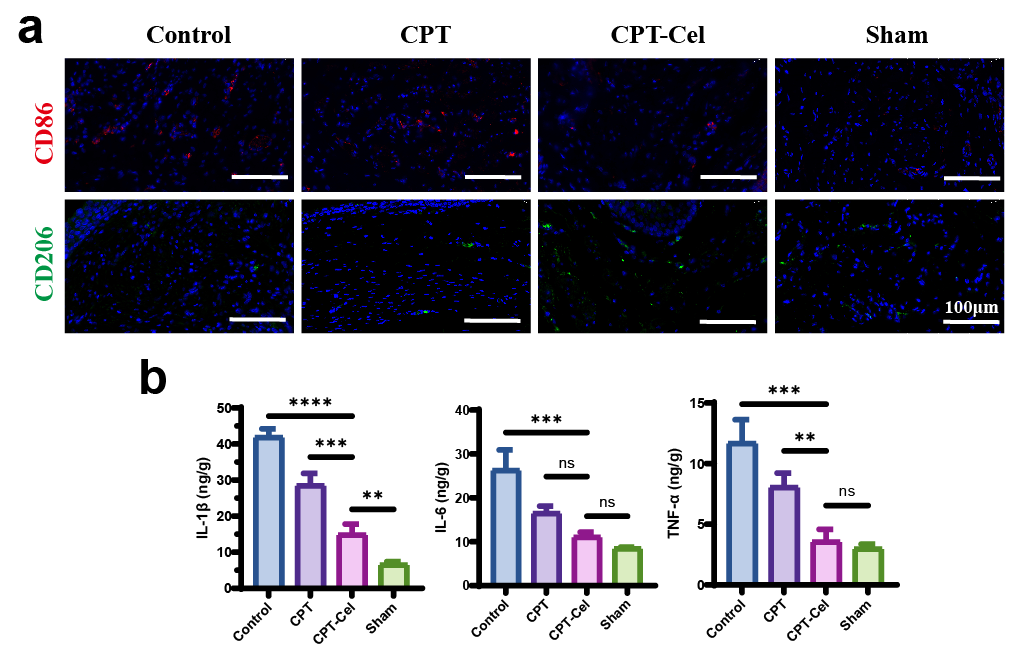


**Fig. S21.** **a)** Immunofluorescence staining for the M1 marker CD86 and the M2 marker CD206 on urethral tissue sections. **b)** ELISA results of IL-1β, IL-6 and TNF-α in urethral tissue homogenates from different groups. One-way ANOVA followed by Dunnett's post hoc test was performed to analyze statistical significance: “ns” indicates no significance, **p < 0.01, ***p < 0.001, ****p < 0.0001.


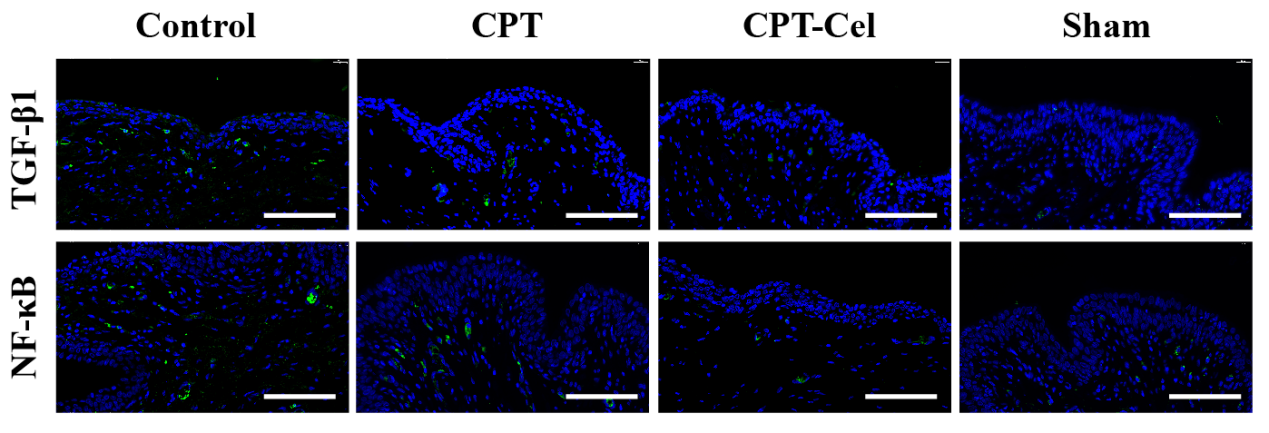


**Fig. S22.** Immunofluorescence staining of TGF-β1 and NF-κB was performed on the 4-week groups to examine the TGF-β and NF-κB signaling pathways.


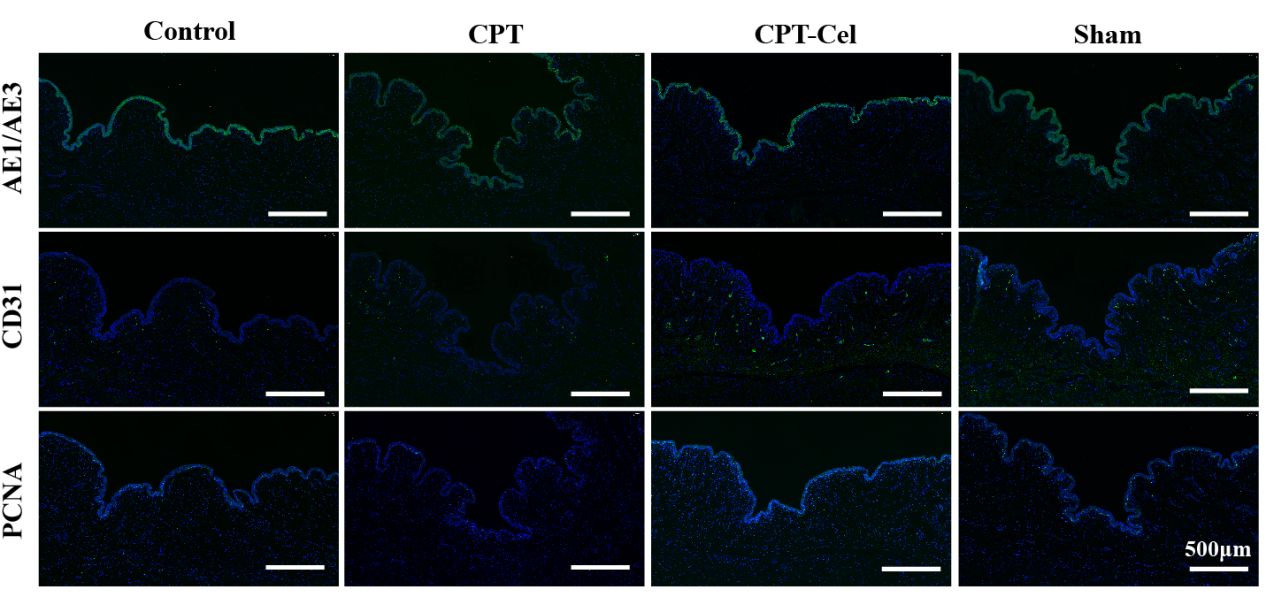


**Fig. S23.** Immunofluorescent staining of AE1/AE3, CD31, and PCNA was performed on the 8-week groups to examine epithelial formation, angiogenesis, and cellular proliferation.

**Table S1.** The antisense and sense primer sequences of different genes for qPCR performance.

| Primer name | Primer sequence (5’-3’) |
| --- | --- |
| NF-kB-S | TGTAACTGCTGGACCCAAGGAC |
| NF-kB-A | CAAATAGGCAAGGTCAGGGTG |
| COX2-S | CGCTCAGCCATACAGCAAAT |
| COX2-A | CTTGAAGTGGGTAAGTATGTAGTGC |
| IL-6-S | GCCACTCACCTCTTCAGAACGA |
| IL-6-A | TCACCAGGCAAGTCTCCTCATT |
| IL-8-S | CAGTTTTGCCAAGGAGTGCTAA |
| IL-8-A | AAACTTCTCCACAACCCTCTGC |
| CXCL1-S | GGGAATTCACCCCAAGAACATC |
| CXCL1-A | GGATGCAGGATTGAGGCAAGC |
| CASP8-S | GGGGTAATGACAATCTCGGACT |
| CASP8-A | GAAGCTCTTCAAAGGTCGTGGT |
| TGFB1-S | CAGCAACAATTCCTGGCGATA |
| TGFB1-A | GCTAAGGCGAAAGCCCTCAAT |
| Smad3-S | CTACCAGTTGACCCGAATGTGC |
| Smad3-A | TCTGTCTCCTGTACTCCGCTCC |
| α-SMA-S | CAATGTCCTATCAGGGGGCAC |
| α-SMA-A | CGGCTTCATCGTATTCCTGTT |
| COL1A1-S | CCCCTGGAAAGAATGGAGATG |
| COL1A1-A | AGCTGTTCCGGGCAATCCT |
| FN1-S | GCATTGCCAACCTTTACAGACC |
| FN1-A | TTGGAAATGTGAGATGGCTGTG |
| GAPDH-S | GGAAGCTTGTCATCAATGGAAATC |
| GAPDH-A | TGATGACCCTTTTGGCTCCC |
